# Uncovering the Key Circuit FOSL2/FOS/EGR3/EGR1, Contributing to the Hyperexcitability of Excitatory Neurons in the Epileptic Temporal Cortex and Hippocampus

**DOI:** 10.3390/ijms27104466

**Published:** 2026-05-16

**Authors:** Jing Chen, Bowen Zhao, Kaiyue Yang, Wanqi Mi, Xiaozhi Huang, Wenqi Jiang, Congxue Hu, Zhenzhen Wang, Yunpeng Zhang, Xia Li

**Affiliations:** 1 College of Bioinformatics Science and Technology, Harbin Medical University, Harbin 150081, China; chenjing1208@hrbmu.edu.cn (J.C.); kzhao52@gmail.com (B.Z.); yangkaiyue0904@hrbmu.edu.cn (K.Y.); miwanqi_9657@163.com (W.M.); huangxiaozhi1108@163.com (X.H.); jwq413078993@163.com (W.J.); hucx1996@hrbmu.edu.cn (C.H.); 2 School of Intelligent Medicine and Technology (Big Data Research Center), Hainan Medical University, Haikou 571199, China; wangzhenzhen@muhn.edu.cn

**Keywords:** epilepsy, excitatory neurons, temporal cortex, hippocampus, neuronal hyperexcitability, single-nucleus RNA sequencing

## Abstract

Epilepsy is mainly characterized by spontaneous seizures caused by hyperactive neural circuits. To delineate the cell-type-specific mechanisms underlying neuronal hyperexcitability, we resolve the hyperexcitability of excitatory neurons across epileptic human brain trans-foci at single-cell resolution to identify the key drivers and potential diagnostic signatures. We constructed a comprehensive atlas encompassing 240,000 cells derived from the temporal cortex and hippocampus, detecting trans-regional cellular and molecular diversity. We further delineated dynamic trajectories, gene expression patterns, and functional reorganization across cell types. Using the LASSO and random forest algorithms, we prioritized the core genes and developed a logistic regression-based diagnostic model. Despite transregional cellular landscape conservation, major cell types varied in abundance. Detailed analysis delineated various excitatory neuron subtypes’ dynamic trajectories, intricate expression, and functional reorganization, with pronounced dysfunction in the posterior hippocampal and temporal cortex networks, indicating hyperactive pro-epileptic effects. Excitatory neurons exhibit an intrinsic ability to autonomously organize themselves into distinct, highly active modules, characterized by a high activation state during epileptogenesis, as illustrated by ten epilepsy-associated functions. Transcription circuits FOSL2/FOS/EGR3/EGR1 promote neuronal hyperexcitability. Integrating epilepsy bulk RNA-seq data, we identified 24 overlapping genes between differential genes and circuit targets. The LASSO and random forest algorithms prioritized three core genes (IL1B, SOCS6, and COL4A1). A logistic regression model based on these three genes showed variable performance, with an apparent AUC of 1.000 in the discovery cohort (GSE256068) and AUCs of 0.974 and 0.722 in and two validation cohorts, indicating the need for further validation. Our study establishes the FOSL2/FOS/EGR3/EGR1 circuit as a master regulator of pathological neuronal hyperactivity across epileptic foci, linking transcriptional activation to network dysfunction. Identifying overactive factors may represent a candidate molecular pathway for future therapeutic exploration against hyperexcitability.

## 1. Introduction

Epilepsy represents a pervasive chronic neurological disorder, pathognomonically defined by aberrant synchronous neuronal firing within neural circuits, clinically manifested as the persistent hyperactivation of excitatory neurons—a pathophysiological state termed hyperexcitability [1,2]. Contemporary pharmacotherapy for epilepsy exerts its effects predominantly through direct suppression of excitatory neuron firing via blockade of voltage-gated sodium, potassium, or calcium channels, or through indirect modulation of neuronal activity by potentiating GABAergic inhibitory neurotransmission. Despite these interventions, approximately one-third of affected individuals develop pharmacoresistant epilepsy, rendering existing treatments ineffective [3,4]. This clinical impasse suggests that current therapeutic strategies largely target downstream effectors of neuronal discharge, leaving unaddressed the upstream molecular mechanisms governing the transition of excitatory neurons from quiescence to sustained hyperexcitability. Elucidating this critical regulatory nexus is therefore imperative for advancing our understanding of epileptogenesis and for identifying novel therapeutic targets.

The functional state of excitatory neurons is not autonomously determined but is subject to intricate modulation by a diverse repertoire of cellular constituents. Inhibitory neurons directly constrain the firing frequency and intensity of excitatory neurons through GABA-mediated neurotransmission [5]. In parallel, glial populations—comprising astrocytes, microglia, and oligodendrocytes—indirectly sculpt the excitatory microenvironment by maintaining ionic homeostasis, providing metabolic support, modulating synaptic architecture, and participating in immune surveillance [6]. During epileptogenesis, a holistic understanding of how these heterogeneous cell types coordinately contribute to, or permit, the transition of excitatory neurons toward a hyperexcitable state at the molecular level remains elusive.

Temporal lobe epilepsy, the most common form of focal epilepsy, typically involves a pathological network encompassing two critical brain regions: the temporal cortex and the hippocampus [7]. These structures are anatomically contiguous, with the hippocampus—situated medially within the temporal lobe—serving as a pivotal modulator of cortical electrical activity; functionally, they collectively constitute the “temporal lobe limbic system”. Nevertheless, marked structural heterogeneity exists between them. The temporal cortex is characterized by a rigorous six-layered neuronal architecture, exhibiting layer-specific distribution and connectivity patterns of excitatory and inhibitory neurons [8]. Conversely, the hippocampus comprises distinct subfields—including the dentate gyrus, CA1, and CA3—each distinguished by differential neuronal proportions, glial reactivity states, and local circuit connectivity [9]. Such structural divergence suggests that aberrant discharges originating in one region may undergo modification in intensity and character by the local microenvironment upon propagation to the other. Under epileptic conditions, normal inter-regional synergy may be disrupted, enabling pathological hyperexcitability to disseminate rapidly along established neural connections, thereby forming ictal propagation pathways. Consequently, delineating the respective roles and interactive mechanisms of the hippocampus and temporal cortex in epileptogenesis is fundamental to understanding the origin and spread of epileptic networks.

Recent advances in single-cell transcriptomics have enabled the dissection of cellular heterogeneity and molecular states within complex brain regions at unprecedented resolution. In this study, we assembled and integrated single-nucleus RNA-seq data derived from the temporal cortex and hippocampus of individuals with epilepsy, aiming to systematically chart the cellular landscape of both regions and uncover the core regulatory mechanisms driving excitatory neuron hyperactivation. We discerned highly conserved cellular compositions across the two regions, yet identified marked regional specificity in the proportional representation and functional enrichment of neuronal and glial populations. Excitatory neurons exhibited pronounced activation states in both the temporal cortex and hippocampus; however, the core biological processes and upstream regulatory determinants involved displayed distinct regional divergence. The differentiation trajectory of excitatory neurons was skewed under epileptic conditions. While the differentiation path of inhibitory neurons remained largely conserved, remodeling of their gene regulatory networks potentially compromised their inhibitory capacity. Analysis of glial cells further revealed a functional division between anterior and posterior hippocampal glia: glial cells in the posterior hippocampus demonstrated coordinated enrichment for synaptic functions, potentially fostering a permissive microenvironment that sustains neuronal hyperexcitability. Integrating these findings, we delineated a core regulatory circuit composed of four transcription factors (FOSL2, FOS, EGR3, and EGR1). The activity of this circuit progressively escalated along the excitatory neuron differentiation trajectory, with its downstream targets significantly enriched for synaptic organization, immune modulation, and multiple established epilepsy risk genes. Moreover, combinatorial assessment of three core downstream genes (IL1B, SOCS6, COL4A1) revealed robust discriminative capacity between epilepsy patients and healthy controls across independent validation cohorts.

In conclusion, this study systematically dissects the transcriptomic signatures of neurons and glia in the temporal cortex and hippocampus of epilepsy patients at single-cell resolution, revealing a core transcriptional circuit that coordinately regulates neuronal hyperexcitability in these brain regions. Collectively, our findings provide novel mechanistic insights into the molecular origins of epileptic hyperexcitability and identify potential molecular targets for developing precision intervention strategies tailored to region-specific pathophysiological features.

## 2. Results

### 2.1. Dissecting Transregional Cellular Composition Independent and Joint-Triggering Epileptic Effects

To investigate cellular and transcriptomic alterations in the temporal cortex (Temp) and hippocampus (Hippo) of human patients with epilepsy, we assembled and integrated publicly available single-nucleus RNA-sequencing datasets. These included cortical samples from nine individuals with epilepsy and ten neurologically normal controls, as well as paired anterior and posterior hippocampal samples from an additional five epilepsy patients. Clinical metadata for all samples is summarized in Table S1. The overall analytical pipeline is summarized in Figure S1. Quantitative assessment using the kBET metric confirmed the absence of significant batch effects within both the temporal cortex and hippocampus datasets (Figure S2A). Following rigorous quality control, 110,169 cortical nuclei and 131,096 hippocampal nuclei were retained for downstream analyses.

**Figure 1:**
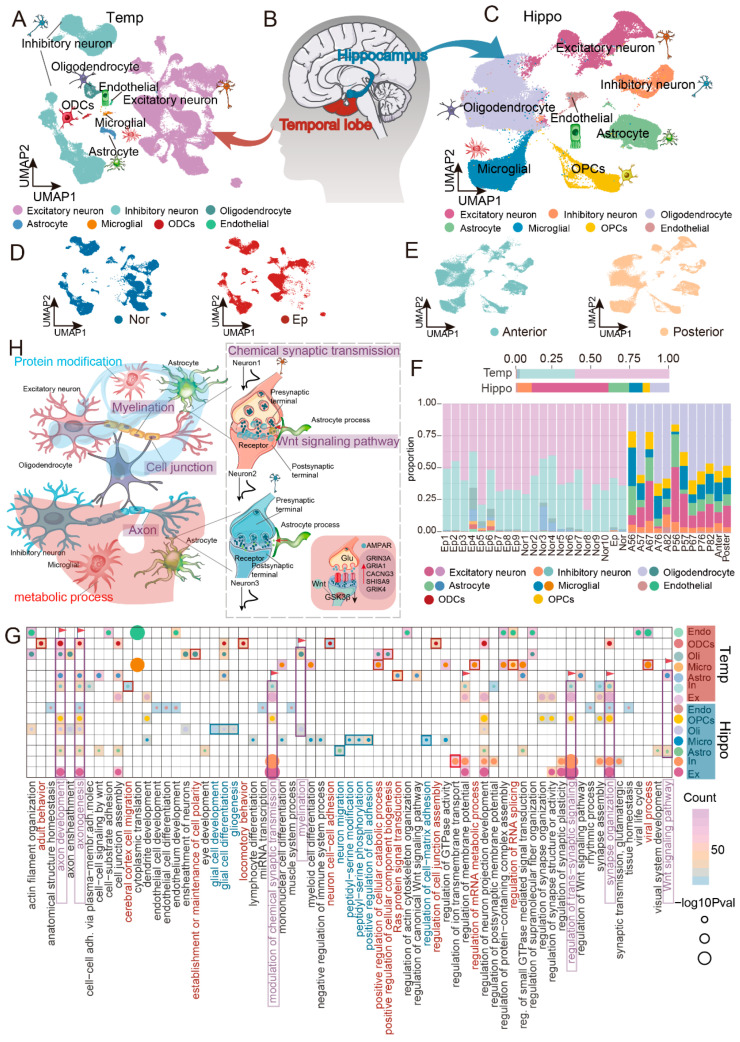
A single-nucleus resolution cross-regional transcriptome reference and cellular function of epileptic human brain. (**A**) Uniform Manifold Approximation and Projection (UMAP) plot demonstrating annotated cell types in temporal cortex, schematic diagram of cells with different morphologies labeling the major cell types identified. ODCs, oligodendrocyte precursor cells; Temp, temporal cortex. (**B**) Schematic illustration of the sampling sites in the temporal cortex and hippocampus, with red representing the temporal cortex and blue symbolizing the hippocampus. (**C**) UMAP plot portraying seven major hippocampal clusters. OPCs, oligodendrocyte progenitor cells; Hippo, hippocampus. (**D**) The cell types UMAP were colored according to cell origin, with normal cells colored in blue and epileptic cortex cells colored in red. Nor, normal temporal cortex; Ep, epileptic temporal cortex. (**E**) UMAP map depicting cells from the anterior and posterior hippocampus, colored by location, with green representing the anterior and yellow the posterior. (**F**) Proportion of different cell types in the temporal cortex (left) and hippocampus (right) of an epileptic brain. Cell colors correspond to Panels (**A**) and (**C**), respectively. “Anterior” refers to the anterior hippocampus; “Posterior” refers to the posterior hippocampus. (**G**) Dot plot demonstrating independent as well as joint-triggering GO terminology among cell types across regions, with purple boxes labeling common GO descriptions, red and blue highlighting cell-specific GO descriptions in temporal cortex and hippocampus. The flags represent biological processes that are co-enriched across different cell types and regions. Ex, excitatory neurons; In, inhibitory neurons; Astro, astrocytes; Micro, microglia; Olig, oligodendrocytes; Endo, endothelial cells. (**H**) Functional diagram illustrating the shared and cell-specific biological functions of glial cells and neurons in the hippocampus and temporal cortex. Purple text indicates shared GO terms, while red and blue text highlight cell-specific GO terms. Schematic cell illustrations in the background mark the major cell types identified in Panels (**A**,**C**). The arrows indicate upregulation or downregulation of gene expression.

Employing a shared nearest neighbor (SNN) algorithm, we initially clustered nuclei from the cortex and hippocampus based on their transcriptional profiles. Using canonical marker genes, we identified highly analogous cellular compositions across both brain regions. These populations included excitatory neurons (Ex), inhibitory neurons (In), astrocytes (Astro), microglia (Micro), oligodendrocytes (Olig), and endothelial cells (Endo) in both the temporal cortex and hippocampus. Notably, while the temporal cortex contained oligodendrocyte precursor cells (ODCs), the hippocampus harbored oligodendrocyte progenitor cells (OPCs) (Figure 1A–C and Figure S2B,C). To systematically evaluate cross-regional similarity while avoiding artifacts from forced integration, we performed independent processing followed by correlation analysis on the transcriptional profiles of all identified cell types instead of co-clustering the two datasets. This conservative approach revealed that corresponding neuronal and glial subtypes from the cortex and hippocampus exhibited striking transcriptional concordance (Figure S2D), indicating that the observed regional differences in the subsequent analyses (e.g., proportions, functional enrichment) are unlikely to be driven by technical batch effects but rather reflect genuine biological heterogeneity.

Upon integrative analysis incorporating cortical data from both epileptic and control individuals, we observed broadly similar cellular landscapes between the two conditions (Figure 1D). Likewise, cell type compositions in the anterior and posterior hippocampus of epilepsy patients were largely superimposable (Figure 1E), indicating that disease state and spatial positioning do not fundamentally reconfigure the core cellular repertoire. Nevertheless, quantitative comparisons of cellular proportions revealed distinct compositional heterogeneity (Figure 1F). Relative to control samples, epileptic cortical tissue exhibited an apparent increase in the frequency of microglia and cells of the oligodendrocyte lineage, accompanied by a corresponding reduction in neuronal fractions, suggesting transcriptomic-level shifts in cortical cellular composition under epileptic conditions. Within the hippocampus, although anterior and posterior subregions harbored identical cell types with broadly similar glial proportions, neuronal populations displayed notable regional disparities: excitatory neurons appeared more abundant in the posterior hippocampus, whereas inhibitory neurons appeared enriched anteriorly. To evaluate these cell-type proportion differences, we applied scCODA, a Bayesian compositional regression model (Table S2). In the temporal cortex, microglia (control effect = −0.29; log2-fold change = −0.49) and oligodendrocyte lineage cells (control effect = −0.31; log2-fold change = −0.52) showed an increase in epilepsy relative to controls, whereas excitatory neurons (control effect = 0.06; log2-fold change = 0.01) showed a decrease. In the hippocampus, scCODA indicated a tendency toward enrichment of excitatory neurons in the posterior region (posterior effect = 0.43; log2-fold change = 0.54) and enrichment of inhibitory neurons in the anterior region (posterior effect = −0.07; log2-fold change = −0.20).

Distinct cell types are likely to exert divergent contributions during epileptogenesis, either facilitating or constraining seizure activity [10]. To delineate signaling pathways potentially underlying seizure generation, we characterized differentially expressed genes (DEGs) across cell populations within each brain region, thereby resolving both cell-type-specific pathways and those shared across cellular subtypes (Figure 1G). Our analysis revealed that even corresponding cell populations assume region-dependent functional identities depending on their localization (cortical versus hippocampal). For instance, microglia residing in the temporal cortex exhibited elevated expression of genes predominantly enriched in metabolic regulation, particularly positive regulation of cellular catabolic processes and regulation of mRNA metabolic processes. This finding points to the existence of an immunopathological microenvironment potentially governed by hyperactivated microglia within the cortical region. In stark contrast, hippocampal microglia shifted functionally toward regulation of cell–matrix adhesion and peptidyl-serine modification, a post-translational modification frequently implicated in modulating protein function. Beyond glial regional specialization, we identified a shared functional signature among neurons across both structures: neurons from both the temporal cortex and hippocampus were profoundly engaged in the regulation of modulation of chemical synaptic transmission. Notably, within the temporal cortex of epileptic individuals, we observed significantly upregulated expression of multiple AMPA receptor auxiliary subunits (e.g., GRIK4, SHISA9, CACNG3) and additional glutamate receptor subunits (e.g., GRIA1, GRIN3A) (Table S3). As AMPA receptors constitute pivotal mediators of fast excitatory synaptic transmission, their dysfunction is intimately linked to neuronal hyperexcitability, implicating aberrant AMPA receptor-mediated signaling in epileptic pathophysiology. Glycogen synthase kinase 3β (GSK3β) has previously been shown to exert antiseizure effects through modulation of the synaptic AMPA receptor pool, and the AMPA receptor antagonist perampanel is already an established clinically approved antiseizure medication [11]. Accordingly, dysregulation of AMPA receptor-mediated synaptic transmission emerges as a critical nexus in epilepsy pathogenesis.

The functional integrity of neurons is inextricably dependent upon the elaborate support provided by glial cells. Astrocytes in both brain regions were significantly associated with the Wnt signaling pathway; active Wnt/β-catenin signaling serves as a master regulator of adult hippocampal neurogenesis, governing neural stem cell proliferation and neuronal fate specification within the dentate gyrus [12]. Critically, glial cells and neurons collaboratively orchestrate key processes including cell junction assembly, regulation of synapse structure or activity, axonogenesis, and axon development. As an illustrative example, a single astrocyte can contact and sustain millions of synapses—not merely sensing and adhering to synaptic structures but also recycling neurotransmitters through glutamate transporters, thereby sustaining ongoing synaptic transmission [13]. Disruption of these precisely coordinated glia-neuron interactions may precipitate aberrant synchronous discharges, ultimately driving epileptic activity.

Collectively, we have constructed a single-cell resolution atlas of the temporal cortex and hippocampus from epileptic patients, revealing both conserved cellular compositions and region-specific proportional and functional specializations across these two core brain structures (Figure 1H). Further analyses pinpointed three signaling pathways potentially intimately associated with seizure activity: first, shared dysregulation of AMPA receptor-mediated chemical synaptic transmission in neurons; second, coordinated involvement of both neurons and glia in cell junction assembly; and third, modulation of synapse structure and activity. Together, these findings converge upon “synaptic dysfunction” as a central pathological event. Whether through overactivation of excitatory synapses or impairment of inhibitory synapses, the ultimate consequence is disruption of the pre-existing homeostatic equilibrium within neural networks, culminating in aberrant neuronal discharge.

### 2.2. Excitatory Neurons Exhibit Highly Active State in Separate Epileptic Regions

Accurate assessment of neuronal activation states during epileptogenesis is imperative, given the fundamental role of neuronal activity in sculpting synaptic plasticity and orchestrating neural circuit development. The human cerebral cortex and hippocampus harbor a remarkably heterogeneous repertoire of neurons, each possessing distinct molecular and functional attributes that collectively assemble into intricate neural networks [9]. Consequently, unraveling the neuronal abnormalities underlying epilepsy necessitates an initial, high-resolution dissection of neuronal diversity within the temporal cortex and hippocampus, alongside elucidation of their cross-regional transcriptional relationships.

**Figure 2:**
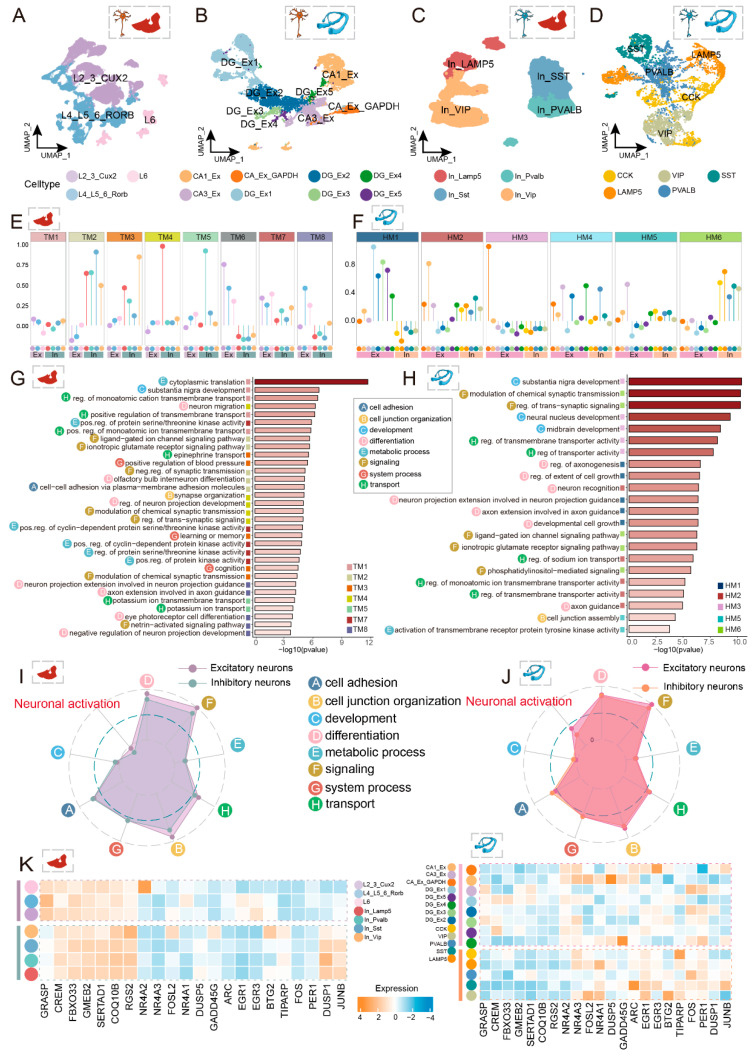
Portrayal of neuronal activity in different epileptic regions. (**A**,**B**) Excitatory neuron subtypes in the temporal lobe (**A**) and hippocampus (**B**), the red shapes depict the temporal lobe cortex, the blue shapes depict the hippocampus, and the cell shapes represent excitatory neurons (consistent with Figure 1). (**C**,**D**) Inhibitory neuron subtypes in the temporal lobe (**C**) and hippocampus (**D**). Colors indicate subpopulations. The cell shapes represent inhibitory neurons (**E**,**F**) AddModuleScore scores for genes in modules for each neuronal cell subtype in the temporal cortex (**E**) and hippocampus (**F**). (**G**,**H**) Function enrichment of overexpressed module genes interrogated by hotspot in the temporal cortex (**G**) and hippocampus (**H**). (**I**,**J**) The radar plots show the differences in EAFs and neuronal activation scores in the temporal cortex (**I**) and hippocampus (**J**); different colors represent excitatory and inhibitory neurons, respectively. (**K**) Average expression of neuronal activation marker genes in individual neuronal cell subtypes in the temporal cortex (left) and hippocampus (right).

We identified both excitatory and inhibitory neuronal populations within these two brain regions and subsequently undertook their subtyping. The classification of excitatory neurons adhered rigorously to their anatomical provenance. Within the six-layered temporal cortex, excitatory neurons were partitioned into three principal subtypes based on layer-specific marker gene expression: superficial layer L2_3_CUX2, middle layer L4_5_RORB, and deep layer L6 (Figure 2A and Figure S2E). Intriguingly, the proportion of cells originating from epileptic tissue samples exhibited a progressive increase with cortical depth (Figure S2F), suggesting that deep-layer neurons may be more actively engaged in epileptic pathophysiology.

Hippocampal excitatory neurons were categorized according to their anatomical subfield origin [14]: the dentate gyrus (DG) and cornu ammonis (CA), yielding five distinct DG subtypes (DG_Ex1–DG_Ex5) and three CA subtypes (CA1_Ex, CA3_Ex, CA_Ex_GAPDH) (Figure 2B and Figure S2G). These subtypes demonstrated marked differential distribution along the anteroposterior hippocampal axis, with DG_Ex1, DG_Ex3, and DG_Ex4 significantly enriched in the posterior hippocampus (Figure S2H), indicating that discrete hippocampal subregions harbor specialized excitatory circuitries.

Inhibitory neuron classification was primarily guided by neurochemical marker expression. These neurons predominantly originate from the embryonic caudal and medial ganglionic eminences and are typically distinguished by their expression of calcium-binding proteins (PVALB), lysosome-associated membrane protein (LAMP5), and specific neuropeptides (CCK, SST, VIP) [15,16]. We identified four inhibitory subtypes within the temporal cortex: In_LAMP5, In_VIP, In_SST, and In_PVALB (Figure 2C and Figure S2I); and five subtypes within the hippocampus: LAMP5, VIP, SST, PVALB, and CCK (Figure 2D and Figure S2J). Excitatory and inhibitory neurons displayed markedly divergent responses to disease conditions. Whether comparing cortical tissue (epileptic versus control) or hippocampal subregions (anterior versus posterior), the proportional representation of excitatory neuron subtypes exhibited substantial fluctuations, whereas inhibitory neuron subtype proportions remained relatively stable across conditions or anatomical locations (Figure S2K,L). This observation suggests that excitatory neurons occupy a more dynamic and activated state during epileptogenesis.

To further explore this distinction, we computed expression correlations of subtype-specific gene signatures across all neuronal populations derived from both brain regions. Excitatory neurons demonstrated pronounced regional modularity: the three temporal cortex subtypes were highly correlated with one another (correlation > 0.7), and hippocampal CA and DG subgroups respectively clustered internally, forming two independent, highly cohesive transcriptional modules (Figure S2M). In contrast, inhibitory neurons exhibited striking cross-regional conservation, with most subtypes showing high transcriptomic similarity between the temporal cortex and hippocampus (Figure S2N). For instance, SST- and PVALB-expressing inhibitory neuron subtypes clustered together, consistent with their proposed shared developmental origin from the medial ganglionic eminence. Collectively, our findings reveal both conserved and region-specific features governing neuronal cell states within the temporal cortex and hippocampus.

Neuronal function depends upon the coordinated action of gene groups. Accordingly, we employed the Hotspot [17] analysis to systematically identify modular co-expression networks within neuronal subtypes of the temporal cortex and hippocampus, respectively. Within the temporal cortex, we identified eight co-expression modules (TM1–TM8) exhibiting distinct activity patterns across neuronal types; notably, modules TM6 through TM8 displayed heightened activity preferentially in excitatory neurons (Figure 2E). Similarly, in the hippocampus, we uncovered six co-expression modules (HM1–HM6), with HM1–HM3 showing selective enrichment in excitatory neurons (Figure 2F). Gene Ontology enrichment analysis of these modules categorized their functions into eight major epileptogenesis-associated functional (EAF) categories, encompassing cell adhesion, cell junction organization, development, differentiation, metabolic processes, signaling, system processes, and transport. Remarkably, module TM8, which was prominently active in temporal cortex excitatory neurons, was significantly enriched for netrin−activated signaling pathway (Figure 2G). Netrin, a canonical axon guidance cue, whose signaling disruption can lead to aberrant axonal pathfinding and misguided neural connectivity [18], suggesting that excitatory neurons may recruit this pathway during epilepsy-associated neural circuit remodeling. Co-expression modules identified in hippocampal neurons were more frequently associated with fundamental biological processes such as cell growth, signal transduction, and the intricate development of brain structures (Figure 2H).

To directly quantify the degree of neuronal electrical excitability, we employed a transcriptome-based activation scoring strategy. By compiling a set of marker genes known to correlate with heightened electrical activity [19] and scoring each cell accordingly, we found that excitatory neurons in both the temporal cortex and hippocampus exhibited significantly higher activation scores compared to inhibitory neurons (Figure 2I,J). This finding establishes that excitatory neuron hyperexcitability constitutes a cross-regional phenomenon. Integrating these observations with the aforementioned EAF scores revealed that excitatory neurons in the temporal cortex generally displayed elevated scores for functions including differentiation, signaling, transport, and cell junction organization. In contrast, the heightened activation of hippocampal excitatory neurons was primarily characterized by a specific enhancement in signaling activities. These analyses, interrogating both functional module enrichment and activation levels, quantitatively confirm a pervasive and specific state of hyperactivation—”hyperexcitability”—within excitatory neurons residing in epileptic foci.

To further investigate the upstream regulators orchestrating this hyperexcitability, we analyzed the expression patterns of a curated set of transcription factor signature genes associated with enhanced neuronal electrical activity. The key transcription factors driving excitatory neuron activation differ markedly between the temporal cortex and hippocampus (Figure 2K). In the temporal cortex, excitatory neurons highly expressed GRASP and FBXO33. GRASP is a postsynaptic scaffold protein implicated in metabotropic glutamate receptor-dependent synaptic plasticity [20]; FBXO33 has been linked to neural responses during seizure activity [21]. The elevated expression of these factors suggests that hyperactivation of temporal cortex excitatory neurons may involve dysregulation of metabotropic glutamate receptor signaling pathways. In the hippocampus, the predominantly highly expressed drivers were ARC and EGR3. ARC, an immediate early gene, mediates synapse-specific signal transduction and plasticity [22]. EGR3 regulates GABAA receptor subunit expression, thereby influencing neuronal development and epileptic pathophysiology [23].

In summary, we systematically delineated the neuronal subtype composition within the temporal cortex and hippocampus of epilepsy patients and quantitatively characterized the hyperactivated state of excitatory neurons at single-cell resolution. Excitatory neurons in the temporal cortex and hippocampus appear to achieve and sustain their hyperexcitable phenotype through engagement of distinct key molecular nodes. This finding provides novel insights into the region-specific molecular mechanisms governing aberrant electrical activity generation in epilepsy.

### 2.3. Excitatory Neurons Exert Hyperactive Influence in Epileptic Human Temporal Cortex and Hippocampus

We employed pseudotime analysis to reconstruct the developmental trajectories of excitatory and inhibitory neurons within the temporal cortex and hippocampus, respectively. This analytical approach aims to elucidate potential disease-associated alterations in neuronal differentiation paths and accompanying functional maturation programs.

**Figure 3:**
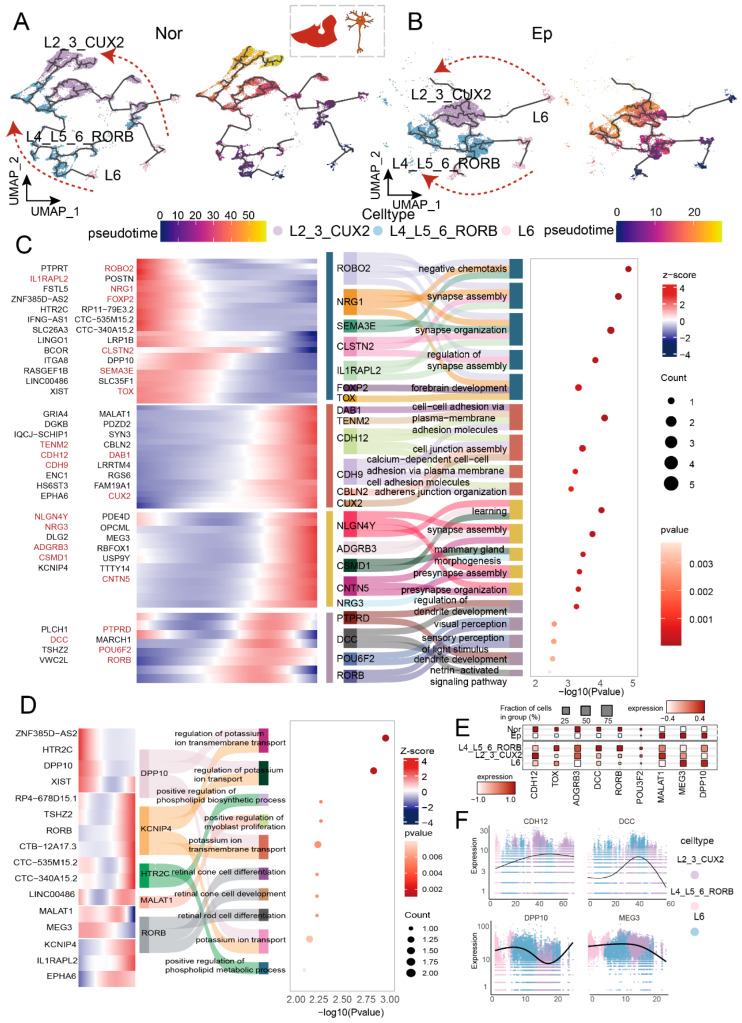
Dynamic differentiation trajectories of excitatory neuron subtype diversity in the temporal cortex. (**A**,**B**) Excitatory neuron subtypes in nonepileptic (**A**) and epileptic (**B**) temporal lobe pseudotime developmental traces. Cells are colored according to cell identity (left) and pseudotime (right), separately. Arrows indicate cell differentiation trends and directions. The red shapes depict the temporal lobe cortex, and the cell shapes represent excitatory neurons. (**C**) Transcriptional transitions of excitatory neurons in the normal temporal lobe with pseudotiming. Left: Heatmap illustrates gene expression associated with cell fate decisions. The rows (genes) of the heatmap are clustered and the columns (cells) are sorted over time. Different stages are labeled with various colors. Middle: Sankey diagram shows the biological processes in which the genes on the left are involved, with different colors pointing to different genes. Right: The dot plot highlights the significance level of the biological processes enriched. (**D**) Transcriptome alterations during the development of epileptic temporal lobe excitatory neurons. Left: Heatmap shows changes in the expression of genes essential for the developmental trajectory. Middle: Sankey diagram demonstrates biological processes of trajectory genes. Right: The dot plot displays the significance level of GO entries. (**E**) Dot plot illustrates the expression levels of excitatory neuron cell fate important genes in various cell subtypes and distinct tissues. (**F**) Expression dynamics of genes activated in developmental trajectories along pseudo-time trajectories.

Within the temporal cortex, excitatory neurons from both epileptic and non-epileptic control tissue followed a conserved overall differentiation trajectory: deep-layer L6 subtypes positioned at the trajectory origin, middle-layer L4_L5_6_RORB subtypes occupying intermediate positions, and superficial-layer L2_3_CUX2 subtypes located at the terminal endpoint (Figure 3A,B). This pattern recapitulates the canonical “inside-out” mode of cortical development, wherein deep-layer neurons (L6) are generated first, followed by later-born neurons migrating past existing layers to ultimately reside in superficial layers (L2/3). Concordant with this migratory sequence, CDH12, which encodes brain cadherin and participates in neuronal migration and synaptic differentiation [24], exhibited enriched expression in superficial L2/L3 layers within normal cortex (Figure 3C).

However, the gene programs and functional modules activated along this shared trajectory diverged fundamentally between epileptic and normal tissue. In non-epileptic cortex, the early trajectory stage (Stage I) was transcriptionally defined by enrichment for genes governing synaptic organization and assembly, including the NRG1/ERBB4 signaling pathway, critical for excitatory neuron migration and synaptogenesis [25,26]. Intermediate stages (II/III) were associated with synaptic assembly and cell junction formation, while the late stage (IV) exhibited significant enrichment for netrin−activated signaling pathway (Figure 3C). Netrin, an axon guidance molecule, coordinately orchestrates signaling and cell adhesion through interaction with DCC receptors, implicating it in neural circuit refinement [18,27]. Collectively, these orderly programs depicted a constructive developmental process aimed at building functional neural networks. In contrast, gene expression along the epileptic cortex trajectory exhibited pronounced dysregulation, with only a limited repertoire of more fundamental and comparatively restricted biological processes being preserved, such as ion transmembrane transport and phospholipid metabolic process (Figure 3D). DPP10, a known modulator of Kv4-mediated A-type potassium currents, regulates neuronal excitability and synaptic integration [28,29,30]. Its role in accelerating the activation and inactivation of Kv4 gating is crucial for preventing pathological hyperexcitability, a hallmark of epilepsy. In our dataset, DPP10 was significantly upregulated in deep-layer L6 subtypes (Figure 3E,F). This functional shift suggests that excitatory neuron maturation in epileptic cortex may be diverted away from constructing fully functional synaptic networks, potentially biasing towards promoting cellular electrophysiological excitability and maintaining basal metabolic homeostasis.

**Figure 4:**
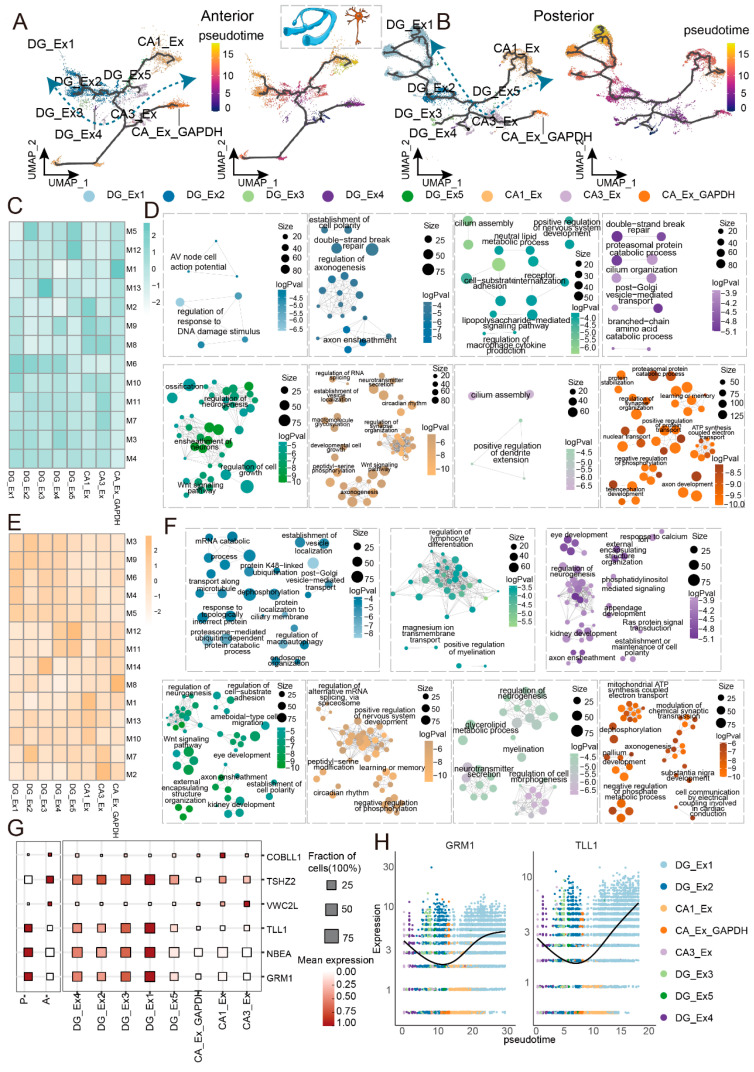
Dynamic differentiation trajectories of excitatory neuron subtype diversity in hippocampus. (**A**,**B**) Excitatory neuron subtypes in anterior (**A**) and posterior (**B**) hippocampus pseudotime traces. Cells are colored according to cell identity (left) and pseudotime (right) separately. Arrows indicate cell differentiation trends and directions. The blue shapes depict the hippocampus, and the cell shapes represent excitatory neurons. (**C**) Co-expression heatmap shows cell-type-specific and shared modules in the anterior hippocampus, where each module displays the aggregated expression values of genes changed along the developmental trajectory involved in each cell subtype. (**D**) Functional network diagram of the top 50 GO entries enriched for genes of the three modules with the highest expression in each anterior hippocampus cell subpopulation. A box qualifies a cell subtype, and the functional color matches the cell type color. (**E**) Heatmap of coexpression for excitatory neuron subtypes in the posterior hippocampus demonstrates trajectory-related specificity to cell type as well as shared modules. (**F**) Functional network map of trajectory-related genes for each excitatory neuron subpopulation in the posterior hippocampus. (**G**) Dot plots display the expression levels of genes associated with functional enrichment and trajectory-related modules of excitatory neuron cells in different cell subtypes and in different positions in the hippocampus. (**H**) Expression dynamics of genes activated in developmental trajectories along pseudo-time trajectories.

The differentiation trajectory of hippocampal excitatory neurons bifurcated into two principal branches (Figure 4A,B). One branch corresponded to the CA region, originating with CA3_Ex, transitioning through CA1_Ex, and terminating at CA_Ex_GAPDH; the other branch corresponded to the DG region, likewise initiating with CA3_Ex but ultimately reaching DG_Ex1. This pattern indicates that distinct hippocampal subfields possess independent differentiation pathways. A recent single-cell atlas of the developing human hippocampus performed pseudotime analysis and explicitly showed that CA1 neurons are more mature than CA3 neurons, and that DG granule cells represent a distinct, later-maturing lineage and our ordering directly recapitulates this developmental hierarchy [9]. To dissect functional changes during differentiation, we clustered genes exhibiting expression variation along the trajectory into discrete modules (Figure 4C) and analyzed the functions of modules highly expressed in each subtype. This analysis revealed markedly distinct functional specializations between anterior and posterior neurons. Anterior-enriched DG subtypes were predominantly associated with regulation of response to DNA damage stimulus, regulation of axonogenesis, lipopolysaccharide−mediated signaling pathway, proteasomal protein catabolic process (Figure 4D). Conversely, the posterior-enriched DG_Ex1 subtype highly expressed PIK3C3 (involved in autophagosome initiation) and PPP3CA/PPP3CC (calcineurin, regulating macroautophagy) [31] (Figure 4E,F; Table S4), suggesting this subtype may function as a stress-associated population maintaining neuronal homeostasis through autophagy. Additionally, the metabotropic glutamate receptor GRM1 was highly expressed in the DG_Ex1 subtype (Figure 4G,H); this receptor modulates intracellular calcium homeostasis through glutamate signaling and is intimately involved in hippocampal long-term potentiation [32]. These findings indicate that posterior hippocampal excitatory neurons integrate more complex stress-responsive and plasticity-related functions during differentiation, potentially contributing to focal hyperexcitability in epilepsy.

In marked contrast to the pronounced functional shifts observed in excitatory neurons, inhibitory neurons exhibited considerable trajectory conservation under epileptic conditions. Within the temporal cortex, the four inhibitory neuron subtypes displayed consistent pseudotime ordering in both epileptic and non-epileptic tissue (Figure S3A,B), clearly reflecting their developmental origins: SST+ and PVALB+ neurons derived from the medial ganglionic eminence positioned at the trajectory onset, while LAMP5+ and VIP+ neurons originating from the caudal ganglionic eminence occupied terminal branches. Interestingly, despite substantial overlap in trajectory-associated genes between disease and control states (Figure S3C,D), the patterns of gene-gene interactions differed markedly (Figure S3E,F). For instance, gene regulatory network inference predicted unidirectional regulation of GRID2 by ASIC2 specifically in epileptic tissue, a regulatory relationship not evident in normal tissue and ASIC2 and GRID2 were upregulated in In_VIP and In_SST subtypes from epileptic samples (Figure S3G,H). ASIC2 encodes an acid-sensing ion channel implicated in neuronal excitation-inhibition imbalance [33]; GRID2 promotes synaptogenesis through the β-NRX1-CBLN1 complex [33,34]. Their dysregulation in epilepsy may compromise inhibitory neuron function, attenuating their regulatory capacity over excitatory neuron activity. Furthermore, elevated ERBB4 expression in In_PVALB may further impair the ability of inhibitory neurons to integrate synaptic signals and execute inhibitory control [35]. Hippocampal inhibitory neurons similarly displayed comparable anteroposterior differentiation trajectories (Figure S4A,B), with largely consistent biological functions across subtypes during trajectory progression (Figure S4C–F). Elevated ASIC2 and ERBB4 expression in the VIP subtype (Figure S4G,H) points towards shared, cross-regional molecular signatures potentially contributing to impaired inhibitory function.

Overall, excitatory neuron differentiation trajectories exhibited pronounced regional specificity in both the epileptic temporal cortex and hippocampus. Cortical excitatory neurons followed an inside-out differentiation pattern, yet displayed substantial functional dysregulation under epileptic conditions; posterior hippocampal excitatory neurons manifested enrichment for stress-associated functions, potentially linked to focal hyperexcitability. By contrast, inhibitory neuron differentiation paths remained relatively conserved, disease-associated alterations in gene interaction patterns may undermine their inhibitory capacity. These findings, viewed through a developmental dynamic lens, illuminate the origins of excitation-inhibition imbalance in epilepsy: not merely excessive excitatory signaling, but also a concomitant, covert failure of inhibitory control.

### 2.4. Glial Cells Mediate Cellular Junction Assembly and Synaptic Organization Functions Along the Hippocampal Anterior and Posterior Axis

Glial cells not only furnish metabolic support to neurons but also actively engage in the formation and maintenance of synaptic structures [36,37]. Given that synaptic dysfunction lies at the core of neuronal hyperexcitability, and given the established role of glial cells in sculpting synaptic architecture, their phenotypic states within the epileptic hippocampus merit in-depth exploration. Focusing on the anterior and posterior hippocampus, we conducted detailed subpopulation identification and functional characterization of astrocytes, microglia, and cells of the oligodendrocyte lineage.

**Figure 5:**
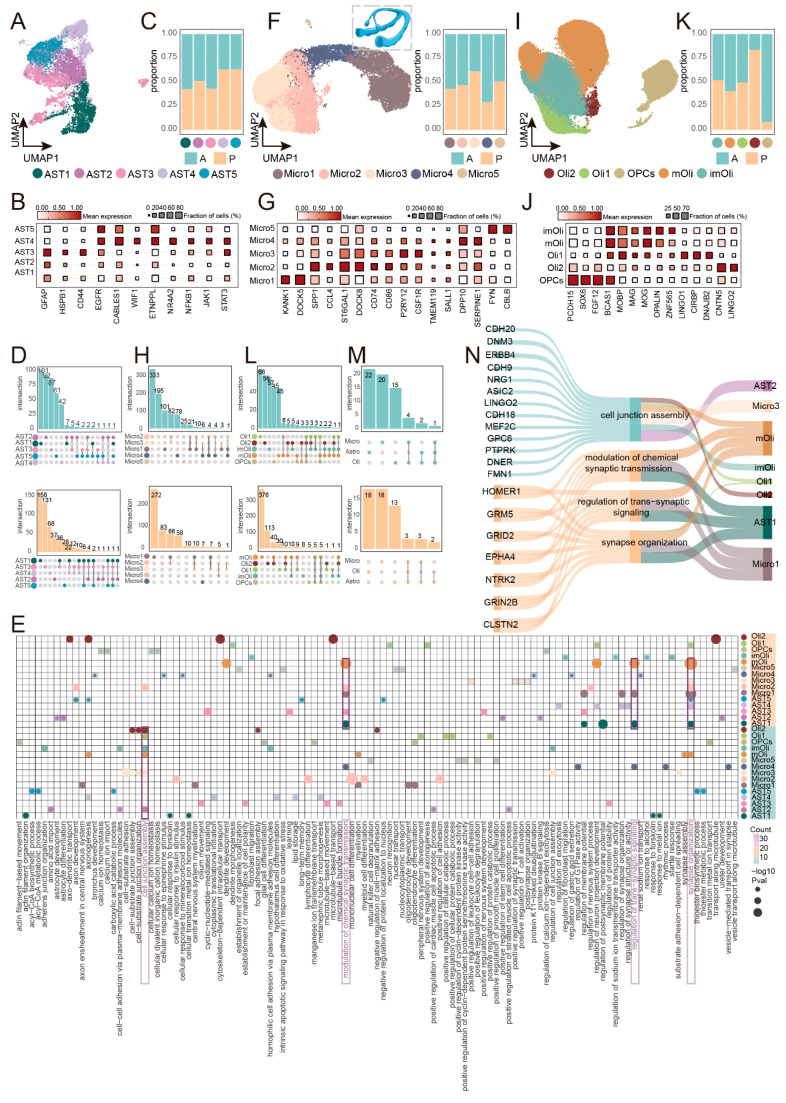
Heterogeneity of transcriptome alterations in subpopulations of glial cells in the anterior and posterior hippocampus. (**A**) Astrocyte subclusters, the blue shape represents the hippocampus. (**B**,**G**,**J**) Dot plots demonstrating the classical marker genes and their expression levels specifically expressed in subclusters of astrocytes (**B**), microglia (**G**), and oligodendrocytes (**J**). (**C**) The proportion of identified astrocyte subclusters at distinct locations in the hippocampus. “A” stands for the anterior hippocampus, and “P” stands for the posterior hippocampus. (**D**,**H**,**L**,**M**) UpSetR plots highlight up- and down-regulated GO terms unique to or shared between subclusters of astrocytes (**D**), microglia (**H**), and oligodendrocytes (**L**), as well as between clusters of these three major types of glial cells (**M**). Bars display the number of GO terms per cluster. Lines between clusters highlight shared GO entries. Green points to GO descriptions that are upregulated in the anterior hippocampus (top) and yellow symbolizes those upregulated in the posterior (bottom). (**E**) Dot plot demonstrating shared as well as specific GO terms across three major types of glial cells, with purple boxes labeling common GO descriptions. (**F**) UMAP map of microglia subclusters, where colors represent cluster logos (left). Bar graph showing the proportion of identified microglia subclusters at different locations of the hippocampus (right). (**I**) UMAP plot of oligodendrocyte subclusters. OPCs: oligodendrocyte precursor cells; imOli: immature oligodendrocytes; mOli: mature oligodendrocytes. (**K**) The proportion of identified oligodendrocyte subclusters in different positions in the hippocampus. (**N**) Riverplot illustrating the relationship between GO terms for astrocytes, microglia with oligodendrocytes acting together at the anterior and posterior ends of the hippocampus and specific genes.

Astrocytes are parenchymal cells distributed throughout the central nervous system, playing indispensable roles in structural support, maintenance of metabolic homeostasis, and synaptic organization. We re-clustered hippocampal astrocytes and identified five transcriptionally distinct subpopulations (AST1–AST5) (Figure 5A,B, Table S5). Among these, AST1, AST2, and AST3 co-expressed the classical reactive marker GFAP, the heat shock protein HSPB1, and the proliferation marker CD44, and were associated with processes such as activity involved in apoptotic signaling pathway and chaperone−mediated autophagy, suggesting they may represent reactive astrocyte states [38]. AST4 exhibited high expression of NR4A2, NFKB1, and JAK1, and was linked to the TGFβ signaling pathway. AST5 was enriched for EGFR, CABLES1, WIF1, and ETNPPL, which implicated in synapse assembly and synaptic structure regulation, consistent with a mature astrocyte phenotype that was relatively more abundant in the posterior hippocampus (Figure 5C). Comparative analysis between anterior and posterior hippocampal regions revealed that even within the same astrocyte subpopulation, functional enrichment diverged markedly along the anteroposterior axis (Figure 5D,E). For instance, genes highly expressed in posterior AST1 were predominantly involved in synaptic-related terms, including regulation of trans-synaptic signaling and synapse organization, encompassing HOMER1, GRM5, and GRID2; whereas anterior AST1 exhibited enrichment for metabolic functions such as response to metal ion and glycolytic process (involving genes like PPARA and MT2A). Posterior AST2 showed enrichment for astrocyte differentiation and gliogenesis, suggestive of possible progenitor-like properties. These findings indicate that astrocytes exhibit pronounced functional regionalization along the anteroposterior axis of the epileptic hippocampus, with posterior subpopulations demonstrating a preferential engagement in synaptic processes.

Microglia, as the brain’s resident immune cells, are equally indispensable for maintaining synaptic homeostasis [36]. We classified them into five subpopulations (Micro1–Micro5) (Figure 5F,G). Micro2 highly expressed SPP1, CCL4, and CD86 and was enriched for immune-related processes such as negative regulation of oxidative stress–induced neuron death and leukocyte proliferation, consistent with an activated microglial phenotype. Micro3 displayed high expression of P2RY12 and CSF1R but low levels of the maturity markers TMEM119 and SALL1, potentially representing an incompletely mature state transitioning towards homeostasis. Micro4, characterized by DPP10 and SERPINE1, was involved in positive regulation of endocytosis and regulation of potassium ion transport, also indicative of an activated microglial subtype. Anteroposterior comparison revealed that microglial functions were more complex and diverse in the anterior hippocampus, with greater functional overlap among subpopulations (Figure 5E,H). For example, the activated subtype Micro4 upregulated peptidyl-serine phosphorylation and regulation of exocytosis anteriorly, while posteriorly it was enriched for protein K11-linked deubiquitination. These regional differences suggest that microglia may participate in immune surveillance and synaptic environment regulation through distinct mechanisms in the anterior versus posterior hippocampus.

Oligodendrocytes are responsible for myelin sheath formation and support axonal metabolism [39]. We identified five subpopulations within the oligodendrocyte lineage: OPCs (oligodendrocyte precursor cells), imOli (immature oligodendrocytes), mOli (mature oligodendrocytes), Oli1, and Oli2 (Figure 5I,J). OPCs highly expressed PCDH15, SOX6, and BCAS1, were enriched for regulation of neuron projection development and axon guidance, and were predominantly localized to the anterior hippocampus (Figure 5K). mOli exhibited high expression of myelin protein genes such as MOBP, MAG, and MOG. Oli2 was characterized by high expression of CNTN5 and LINGO2, and was significantly enriched for synapse assembly, modulation of chemical synaptic transmission, and regulation of trans-synaptic signaling; notably, this subpopulation was primarily concentrated in the posterior hippocampus. Functional comparison along the anteroposterior axis revealed that OPCs upregulated oligodendrocyte differentiation, myelination, and axonogenesis anteriorly, while posteriorly they upregulated ion-related functions such as cellular calcium ion homeostasis (Figure 5E,L). Oli1 and Oli2 posteriorly were enriched for positive regulation of axonogenesis and synapse organization. These findings suggest that cells of the oligodendrocyte lineage assume distinct functional roles along the hippocampal anteroposterior axis, with the posterior-enriched Oli2 subpopulation potentially exerting direct influence on neuronal excitability through modulation of synaptic transmission.

Integrating across the three major glial lineages revealed a principle of spatial functional compartmentalization: in the anterior hippocampus, astrocytes, microglia, and oligodendrocyte lineage cells collectively exhibited enrichment for cell junction assembly, involving genes such as ERBB4, NRG1, and ASIC2; in the posterior hippocampus, they shared enrichment for three intimately interconnected functions: modulation of chemical synaptic transmission, regulation of trans-synaptic signaling, and synapse organization, involving genes including HOMER1, GRM5, and GRID2 (Figure 5E,M,N). The protein products of these genes are established players in synaptic plasticity, glutamate signaling, and the regulation of neuronal excitability [40].

Collectively, these findings underscore that, within the epileptic hippocampus, the function of cell junction assembly in preserving neural network structural integrity and the synaptic modulation functions governing information transmission are not mediated exclusively by neurons. Instead, they are orchestrated by glial populations through regionally coordinated cooperation. The concerted predisposition of posterior glial cells toward synaptic functions may establish a supportive microenvironment that sustains neuronal hyperexcitability through local modulation of the synaptic milieu. This observation suggests that glial functional heterogeneity may represent a cellular substrate predisposing the posterior hippocampus to serve as an epileptic focus.

### 2.5. Revealing Circuit FOSL2/FOS/EGR3/EGR1 Transregional Crosstalk Promoting Excitatory Neuronal Activation

Excitatory neurons exhibited pronounced activation states in both the temporal cortex and hippocampus, prompting consideration of a cross-regional orchestrated regulatory mechanism. Specifically, we investigated whether a shared transcriptional regulatory program coordinates neuronal hyperexcitability during epileptogenesis. To address this question, we constructed cell-type-specific gene regulatory networks in the temporal cortex and hippocampus, respectively.

**Figure 6:**
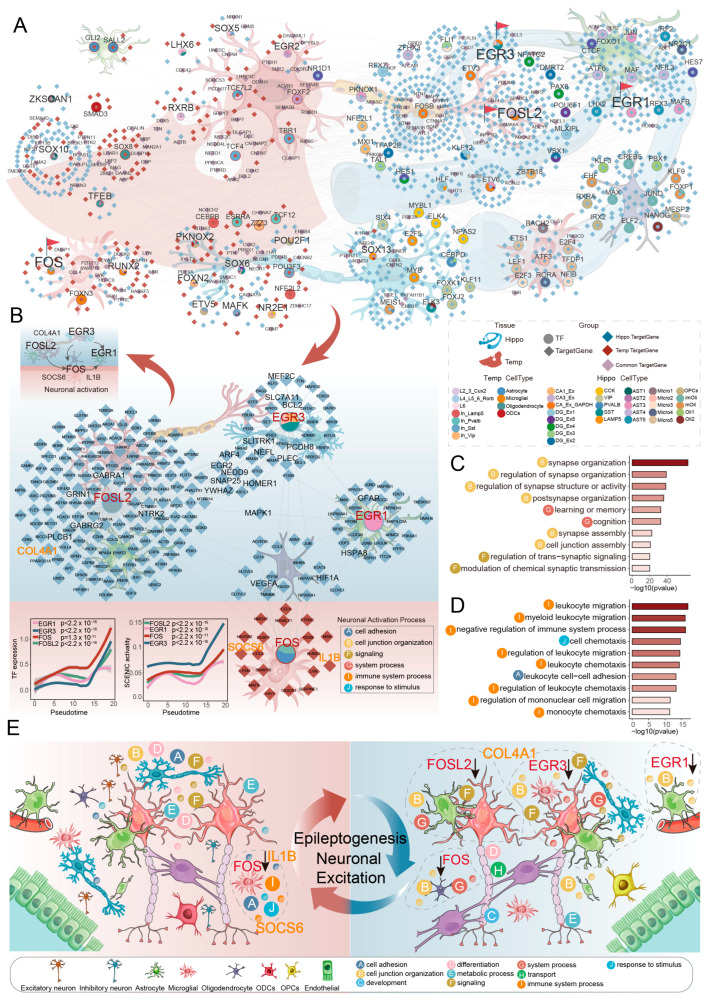
Identification of neuronal activation-associated transregional transcriptional modules. (**A**) Cell type-specific transcriptional regulatory networks are depicted for the epileptic temporal cortex (left) and hippocampus (right). Transcription factors (TFs) are represented by circles, with the inner pie chart color indicating the cell subtype. Target genes (TargetGene) to which the TFs bind are marked with diamonds. TargetGene co-occurring in both regions are indicated in purple, with red diamonds representing TargetGene in the temporal lobe and blue diamonds for those in the hippocampus. The shaded areas at the bottom represent distinct tissues, with blue representing the hippocampus, red representing the temporal cortex, and different shapes symbolizing various cell types. TF, Transcription factor; Temp, temporal cortex; Hippo, hippocampus. (**B**) Subnetworks of modules associated with neuron-activated cross-regional transcriptional circuits (extracted from Panel (**A**)) and their schematic representation (top left); the fitted curve in the bottom left corner illustrates the trends in the expression and activity of key transcription factors over pseudotime. (**C**,**D**) Functional enrichment of transcription factors and their target genes within transcriptional modules in the hippocampus (**C**) and temporal cortex (**D**), yielding five epileptogenesis-associated functional (EAF) categories. (**E**) Illustration of the putative transcriptional regulation process of cell subtype-associated neuronal activation transcriptional modules and their molecular functions in the temporal cortex (left) and hippocampus (right). The arrows indicate the genetic composition of the key circuit, and the different letters represent different Epilepsy-associated functions (EAF) categories.

The complexity of gene regulatory networks within hippocampal neurons substantially exceeded that observed in the temporal cortex. This heightened complexity stemmed from greater network density in the hippocampus, where transcription factors exerted broader regulatory influence by targeting a larger repertoire of genes. Within the hippocampus itself, the transcription factor-target gene interaction network was more densely interconnected in the CA region compared to the dentate gyrus, and the inclusion of glial cells further amplified network complexity (Figure 6A and Figure S5A).

Despite these regional disparities, we identified a set of transcription factors commonly expressed across both brain regions. Among these, members of the FOS family (FOS, FOSB, FOSL2) and the EGR family (EGR1, EGR3) were expressed across multiple cellular subtypes, suggesting that they may serve coordinative functions. To identify the transcription factors directly linked to neuronal activation, we focused on immediate early genes that were both upregulated under epileptic conditions and intimately associated with neuronal electrical activity. Integrating pseudotime analysis revealed that the expression levels of FOSL2, FOS, EGR3, and EGR1 progressively increased along the excitatory neuron differentiation trajectory (Figure 6B). This dynamic expression pattern indicated that the activity of this transcription factor constellation synchronized with the transition of neurons from quiescence toward hyperexcitability, reinforcing their centrality in establishing and sustaining neuronal hyperexcitability. To assess whether the observed expression trends depend on the specific root assignment, we therefore performed a formal sensitivity analysis by re-running pseudotime inference with alternative root assignments, using the average expression of these four immediate early genes (IEGs) as an activity-readout. For the temporal cortex, the choice of L6 as the trajectory origin is strongly supported by the canonical ‘inside-out’ developmental sequence of the mammalian cortex, where deep-layer neurons are generated first [41,42,43]. Using the correct deep-layer root (L6), the average expression of the four IEGs showed a significant positive correlation with pseudotime (non-epileptic: *p*< 2.2 × 10^−16^; epileptic: *p* = 1.8 × 10^−6^; Figure S6A,B). When the superficial layer L2_3_CUX2 (an incorrect root representing the end of inside-out development) was chosen, the correlation became negative in non-epileptic cortex (*p* < 2.2 × 10^−16^) and non-significant in epileptic cortex (*p* = 0.28). These results confirm that only the biologically correct root (L6) yields a pseudotime ordering consistent with the established cortical development sequence. For the hippocampus, we tested CA3_Ex (original), CA1_Ex, and DG_Ex1 as roots. The selection of CA3_Ex as the origin is supported by its highest transcriptomic correlation with cortical L6 neurons (Spearman’s R = 0.81), as well as by the expression gradient of DG maturation markers PROX1 and LGI1 (lowest in CA3_Ex, highest in DG_Ex1) and the decreasing trend of the GOBP_GLUTAMATERGIC_NEURON_DIFFERENTIATION module score along pseudotime (Figure S6C,D). Under the most biologically justified root (CA3_Ex), the average IEG expression correlated positively with pseudotime (*p* < 2.2 × 10^−16^; Figure S6E). Choosing DG_Ex1 (a terminally differentiated granule cell) as the root gave a weaker positive correlation. Moreover, setting DG_Ex1 as the root caused PROX1 and LGI1 to decrease along pseudotime, contradicting their known roles as markers of granule cell maturation. Collectively, this sensitivity analysis demonstrates that only the developmentally correct roots (L6 for cortex, CA3_Ex for hippocampus) produce a pseudotime ordering consistent with independent molecular markers and known developmental principles. The progressive increase in the FOSL2/FOS/EGR3/EGR1 circuit along excitatory neuron differentiation is thus robust when the biologically appropriate differentiation direction is used.

We thus defined these four transcription factors, FOSL2, FOS, EGR3, and EGR1, as a core transcriptional circuit driving neuronal activation during epileptogenesis. The significance of this regulatory circuit was further underscored by its downstream target genes. Notably, multiple targets within this circuit, including GABRA1, GABRG2, MEF2C, BCL2, GFAP, and HIF1A, represent established epilepsy risk genes cataloged in the Online Mendelian Inheritance in Man (OMIM) database (Figure 6B). Previous studies have demonstrated that the transcription factor FOS plays critical roles in neuronal plasticity, neural network formation, and immune response modulation [44,45]. Meanwhile, EGR3 serves as a key regulator of GABRA4, influencing neuronal development and epileptogenesis [23]. These observations indicate that this core transcriptional circuit directly participates in shaping epileptic pathophysiology through regulation of a panel of confirmed epilepsy-associated genes.

Within the hippocampus, the downstream targets of this circuit were significantly enriched for functions such as cell junction assembly and regulation of trans-synaptic signaling (Figure 6C). In the temporal cortex, in addition to synapse-related functions, enrichment extended to negative regulation of immune system process within microglial populations (Figure 6D). Collectively, these findings suggest that the same core transcriptional circuit may contribute to the hyperexcitability of excitatory neurons across different brain regions by influencing distinct cellular populations and biological processes. In the hippocampus, it may more directly drive synaptic dysfunction, whereas in the temporal cortex, it may additionally engage in orchestration of the neuroimmune microenvironment. Thus, through regulating a suite of established epilepsy-associated genes, this core circuit participates directly in synaptic dysfunction and neuroimmune microenvironment remodeling, thereby molecularly linking neuronal hyperexcitability to epileptic pathophysiology.

To explore the clinical relevance of this neuronal activation circuit, we integrated bulk RNA-sequencing data from epileptic patients at the tissue level. In the GSE256068 cohort, we identified 1917 differentially expressed genes, comprising 989 upregulated and 927 downregulated genes (Figure S5B). These differentially expressed genes were significantly enriched in pathways such as Neuroactive ligand−receptor interaction, Neuroactive ligand signaling, and positive regulation of cell activation (Figure S5C,D), providing tissue-level corroboration of sustained neuronal hyperactivation during epileptogenesis. To bridge the core regulatory mechanisms identified at single-cell resolution with differentially expressed genes observed at the tissue level, we intersected these differentially expressed genes with the downstream targets of the FOSL2/FOS/EGR3/EGR1 transcriptional regulatory circuit, yielding 24 candidate genes closely associated with neuronal activation (Figure S5E). Through two machine learning algorithms, LASSO and random forest, we ultimately prioritized three core genes: IL1B, SOCS6, and COL4A1 (Figure S5F–I). A logistic regression diagnostic model constructed based on these three genes demonstrated robust discriminative performance in the discovery cohort (AUC = 1.000) (Figure S5J). External validation in two independent cohorts gave AUCs of 0.974 (GSE139914, individual gene AUCs: IL1B = 0.667, SOCS6 = 0.968, COL4A1 = 0.830) and 0.722 (GSE140393, individual gene AUCs: IL1B = 0.639, SOCS6 = 0.546, COL4A1 = 0.630) (Figure S5K,L). Notably, in GSE140393, the individual AUC for SOCS6 was only 0.546, indicating poor predictive performance for this gene in that dataset. The reduced performance across both the combined model and individual genes in GSE140393 suggests that the three-gene signature has limited generalizability, likely due to the small sample size (12 epilepsy vs. 9 controls) and inter-study heterogeneity. The heterogeneity in validation performance can be partly explained by differences in dataset characteristics: GSE139914 and GSE140393 are both derived exclusively from temporal lobe tissue but differ substantially in sample size (8/39 vs. 12/9) and epilepsy subtype annotation. A comparative summary of all three datasets is provided in Table S6. Therefore, while the signature captures some biological signal related to the core transcriptional circuit, its diagnostic utility is not robust across all settings. These three genes should be considered exploratory transcriptional correlates rather than a validated clinical diagnostic tool, and future prospective studies with larger, more homogeneous cohorts are required to assess their true predictive value.

In summary, we have identified a transcriptional core circuit FOSL2/FOS/EGR3/EGR1 at single-cell resolution that coordinately drives neuronal hyperexcitability (Figure 6E). The activity of this circuit synchronizes with the state transition of excitatory neurons, and its downstream targets are enriched for synaptic organization and immune modulation functions, encompassing multiple established epilepsy risk genes. Serving as a key nexus linking distinct brain regions and coordinating neuronal hyperexcitability with associated pathological microenvironments, this circuit provides a theoretical basis and specific molecular targets for deepening our understanding of epilepsy pathogenesis and for developing novel intervention strategies.

## 3. Discussion

Epilepsy is a chronic neurologic disorder with wide-ranging effects, and temporal lobe epilepsy is still the more common form in adults. Temporal lobe epilepsy is closely interrelated with pathological features of hippocampal sclerosis, cellular variants mediating Ras/Raf/MAPK signaling enriched in the hippocampus may be at risk for promoting the development of refractory epilepsies [46].

While traditional approaches such as medication or surgery can permeate the brain and provide widespread treatment, they lack the precision needed to target and modulate specific overactive, abnormal brain regions. Gene therapy, on the other hand, has emerged as a promising strategy, offering the potential to regulate hyperactive brain areas by delivering functional genes capable of controlling spontaneous seizures. However, a significant challenge remains: the inability of current gene therapies to effectively differentiate between “healthy” and “overactive” neurons [47,48]. This limitation can result in the unintended overactivation of “healthy” neurons, thereby disrupting normal brain function and potentially leading to adverse effects. Consequently, there is a critical need for the development of more refined neuronal overactivity genes that can precisely target pathological neuronal activity without compromising the integrity of unaffected brain regions.

At the transcriptomic level, the occurrence of temporal lobe epilepsy implies that neuronal circuits are affected by overexcited neuronal and non-neuronal cellular activity. We found that the cellular ecosystems in the epileptic temporal cortex and hippocampus were similar, both being rich in excitatory and inhibitory neurons, astrocytes, microglia, oligodendrocyte-spectrum cell subtypes, and that these two key epileptic brain regions shared common neuronal and glial cell features and jointly trigger cell junction assembly, regulation of synapse structure or activity, and modulation of chemical synaptic transmission these three underlying seizure-triggering signaling pathways. These signaling pathways require the tacit and precise cooperation of glial cells and neurons.

In our subsequent analysis, we precisely quantified neuronal activation across different epileptic foci at the single-cell level, revealing a pronounced elevation in the activation of excitatory neurons compared to inhibitory neurons. Additionally, we curated a comprehensive list of epilepsy-related biological functions, encompassing processes such as cell adhesion, cell junction organization, development, differentiation, metabolic pathways, signaling, systemic processes, and transport. This framework enabled a more granular dissection of the specific cellular processes driving neuronal hyperactivity within epileptic foci. Notably, the biological functions that promote excitatory neuronal activity were found to vary across different epileptic regions, each governed by distinct transcriptional regulators.

We systematically analyzed the developmental trajectories of excitatory and inhibitory neurons in the temporal cortex and hippocampus to reveal the diversity and heterogeneity of neuronal cell subtypes. During cell development, excitatory neuron subtypes were found to be more dysfunctional in the epileptic temporal lobe cortex relative to normal tissues, and on the contrary inhibitory neurons had remarkably identical biological functions in different disease states, revealing that excitatory neuron activity was hyperactive and inhibitory neuron activity was relatively stable during epileptogenesis, which suggested that an excitatory-inhibitory imbalance contributes to epileptogenesis.

Subsequently, we delved into the transcriptional regulation of neuronal activation, with the objective of identifying key factors that could selectively target and modulate neurons implicated in the initiation of epileptic seizures. Activity-dependent promoters, particularly those associated with immediate early genes (IEGs), exhibit rapid responsiveness to heightened neuronal activity. Given that seizures typically manifest with sudden onset and termination, often occurring in clusters with intervals that align with the dynamics of IEG expression [49], we identified the FOSL2/FOS/EGR3/EGR1 transcriptional modules. These modules display a progressive increase in transcriptional activity concurrent with neuronal differentiation. Notably, the biological processes that potentiate neuronal activity vary significantly across different epileptic foci. For instance, in the temporal cortex, the predominant process is immune-responsive, closely linked to microglial activation. Epileptic neuronal hyperexcitability triggers microglial activation and drives the expression of CD39 through the recruitment of cAMP-responsive element-binding proteins. In turn, activated microglia inhibit neuronal hyperexcitability in a CD39-dependent manner [50]. Our study extends previous single-nucleus transcriptomic analyses of epileptic tissue [51,52] by uncovering a cross-regional transcriptional circuit (FOSL2/FOS/EGR3/EGR1) that is progressively activated along excitatory neuron differentiation and whose downstream targets are enriched for epilepsy-associated genes. This finding may inform future strategies for targeted modulation of hyperexcitable brain regions.

Several limitations of this study should be acknowledged. First, the hippocampal dataset was derived from five individuals with epilepsy and lacked healthy control samples. Importantly, our primary focus was on comparing the anterior versus posterior hippocampus in a paired design in which each patient contributed both regions. This design effectively reduces inter-individual variability (e.g., in genetic background, age, and sex) and provides a robust internal control for detecting anteroposterior molecular differences. This design allows us to confidently identify anteroposterior molecular heterogeneity within the epileptic hippocampus. However, the absence of non-epileptic samples means we cannot attribute these regional differences solely to epilepsy, as they may also reflect normal human hippocampal specialization. Second, the small sample size (*n* = 5) raises the possibility that certain findings, particularly those related to rare cell subpopulations or donor-enriched clusters, may be influenced by individual variation. Detailed clinical covariates, such as precise epilepsy etiology, time from last seizure to tissue resection, and antiepileptic drug regimen at the time of surgery, were not available for the public datasets used in this study due to patient anonymization and the retrospective nature of the data. These factors may influence gene expression and cellular states. Future studies with larger cohorts and inclusion of healthy control hippocampal tissue are required to validate our conclusions. Although our integrated bioinformatic analysis identifies the FOSL2/FOS/EGR3/EGR1 circuit as a candidate driver of neuronal hyperexcitability, this study is purely computational and lacks direct experimental perturbation. The predicted regulatory relationships and the causal role of these transcription factors in promoting epilepsy-associated hyperexcitability remain unvalidated. Nevertheless, the robustness of our core conclusion regarding the FOSL2/FOS/EGR3/EGR1 circuit is independently supported by a recent study, which identified a similar immediate early gene (IEG)-centered activation module in healthy human hippocampus and an electrically evoked seizure (ECS) mouse model [53]. Future studies should employ functional approaches—such as CRISPR-mediated knockdown or overexpression of FOSL2, FOS, EGR3, and EGR1 in human induced pluripotent stem cell (iPSC)-derived neuronal cultures or in vivo rodent seizure models—to test whether manipulating this circuit can attenuate hyperexcitability. Electrophysiological recordings (e.g., patch-clamp or multi-electrode arrays) will be essential to directly measure changes in neuronal firing. Such experiments will be critical to establish causality and to evaluate the genuine therapeutic potential of targeting this transcriptional circuit.

## 4. Materials and Methods

### 4.1. Multi-Sample Epilepsy Single-Nucleus Sequencing Dataset Report

We retrieved single-nucleus sequencing datasets of hippocampus and temporal cortex tissues of epileptic patients from the GEO database (https://www.ncbi.nlm.nih.gov/geo/, GSE160189, accessed on 10 September 2023) and GitHub (https://github.com/, accessed on 15 October 2023), a hosting platform for open-source and private software projects (https://github.com/khodosevichlab/Epilepsy19). The temporal lobe cortex dataset contained 9 epileptic patients whose MRI showed abnormal symptoms linked to epileptic pathology and 10 non-epileptic patients, for a total of 21 samples [54]. The hippocampal tissues were all derived from 5 epileptic patients with non-temporal sclerosis or other hippocampal lesions, each of which was removed based on neuroanatomical regions of the hippocampus anterior (Anterior, represented by “A”) and posterior (Posterior, represented by “P”), for a total of 10 samples.

### 4.2. Quality Control, Cell Clustering and Major Cell Type Identification of snRNA-Seq Data

The expression matrix for each sample was loaded as a Seurat object (v 4.1.1) [55], and in order to exclude low-quality and bimodal cells, we performed quality control on the temporal cortex-independent and hippocampus-independent datasets using two strategies: firstly, for each sample, the genes were present in at least 3 cells, and those cells that expressed more than 6000 or less than 200 genes in the temporal cortex dataset were filtered, while cells expressing 200–4000 genes were retained in the hippocampus dataset. Quality control thresholds for nFeature_RNA were determined independently for each dataset based on the empirical distribution of gene counts per cell, with the goal of removing low-quality cells and potential doublets (>6000 genes for cortex; >4000 genes for hippocampus). The different upper thresholds reflect intrinsic differences in sequencing depth and library complexity between the two datasets. Secondly, those cells that expressed more than 10% of the mitochondrial genes were excluded to maximize the preservation of the dataset characteristics and quality. Overall, we captured 110,169 temporal cortex cells and 131,096 hippocampal cells for further analysis.

The temporal cortex and hippocampus datasets were derived from two independent published studies (GSE160189 and a GitHub repository, respectively). To avoid potential over-correction that might eliminate genuine regional biological variability, we deliberately processed the two datasets separately rather than performing forced integration (e.g., via Harmony or CCA). For the cortical dataset, epilepsy and control samples were processed using the same cDNA library preparation protocol, minimizing technical batch effects. Control samples had very low post-mortem intervals (<1 h), the original study reported good integration across samples with no observable experiment-batch effects after using Conos [54]. For the hippocampal dataset, the original authors applied Seurat’s IntegrateData (CCA) and regressed out batch, age, sex, and 10X chemistry version. They also removed sex chromosomes from the analysis and found no significant sex effect [14]. To exclude age- and sex-driven effects, we reran the scCODA [56] models with age group (Adult, 18–55 vs. Old, >56) or sex as the sole covariate. No cell type exhibited a credible association with either variable (all final parameter = 0), confirming that the observed condition-dependent changes are not confounded by age or sex (Table S2). To quantitatively assess the mixing of samples from different batches, we applied the kBET (K-nearest neighbor Batch Effect Test) using the R package kBET [57]. The test computes the acceptance rate of local neighborhood mixing across batches, with rates above 0.8 (80%) considered as indicative of effective batch effect removal. Analyses were performed on the UMAP embeddings of both the temporal cortex and hippocampus datasets, using default parameters (k0 = 100, n_repeat = 10). Consequently, no additional batch correction was applied.

We performed a principal component analysis based on the first 2000 highly variable genes identified by the “FindVariableFeatures” function and downscaled the dataset. Based on the JackStraw analysis, the optimal number of principal components (1:30) for cluster analysis was determined in the temporal cortex dataset based on the elbow map, and the top 25 principal components were selected in the hippocampus. Then, we clustered using a clustering algorithm based on the modular optimization of the Shared Nearest Neighbors (SNN), a neighborhood map was obtained, for which we conducted the dimension reduction and visualization using the Uniform Mobility Approximation and Projection (UMAP), individually. We employed a resolution of 0.6 to cluster the cells. Each cell type marker gene was ascertained by the FindAllMarkers() program, and the Wilcoxon rank sum test was utilized to obtain differential *p*-values for the genes, with the specific criteria set to pct = 0.1, logfc. threshold = 0.25, and only.pos = T. Cell type differentially expressed genes were combined with classical cell markers in CellMarker 2.0 to annotate the major cell types individually. A total of eight cell types were identified in both tissues: excitatory neurons (Ex), inhibitory neurons (In), astrocytes (Astro), microglia (Micro), oligodendrocytes (Olig), oligodendrocyte precursor cells (ODCs), oligodendrocyte progenitor cells (OPCs), and endothelial cells (Endo).

To statistically evaluate cell-type proportion differences while accounting for the compositional nature of single-cell data, we applied scCODA [56]. For the temporal cortex dataset, we modeled the effect of disease status (epilepsy vs. control) as the sole covariate; for the paired hippocampal dataset, we modeled the effect of anatomical location (anterior vs. posterior). All models were run with default priors. The direction of change was determined by the Final Parameter value (positive = increased relative to the reference condition; negative = decreased).

### 4.3. Cell Subpopulation Re-Annotation

We reclustered neuronal cells and glial cells in both regions. The analysis was first carried out following the Seurat pipeline process, for excitatory neurons, in the temporal cortex we took a resolution of 0.8 to cluster the cells, identifying three main categories based on the layer position-specific expression of genes L2_3_CUX2 (LAMP5, CUX2), L4_5_RORB (RORB, COBLL1, TOX), L6 (TLE4, NTNG2). Whereas the clustering resolution in the hippocampus was set to 0.6, we classified into DG and CA classes based on the validated dentate gyrus marker genes (MAML2, SEMA5A) as well as CA marker genes (SV2B), and combined with the differential genes among the cellular subtypes, we obtained the five major DG subgroups DG_Ex1 (SLC47A1, COLEC12), DG_Ex2 (MAML2, SEMA5A), DG_Ex3 (SERPINE1), DG_Ex4 (LHFPL3, TMEM132C), DG_Ex5 (SLC14A1, TNC) and three CA classes CA1_Ex (PID1), CA3_Ex (HS3ST4), CA_Ex_GAPDH (GAPDH). For inhibitory neurons, since most of the inhibitory neurons in the temporal cortex and hippocampus are of similar origin, we categorized and annotated inhibitory neurons in the temporal cortex and hippocampus separately in view of the known reference categorization strategies, with four subtypes, In_LAMP5, In_VIP, In_SST, and In_PVALB acquired in the temporal cortex, and five cellular subtypes, LAMP5, VIP, SST, PVALB, and CCK, recognized in the hippocampus.

Given that the temporal cortex dataset was focused on neuronal investigation during the sequencing process and had a low content of neuroglial cells, only the three major types of neuroglial cells in the hippocampal tissue were analyzed (astrocytes, microglia, and oligodendrocyte lineage cells) in the present study. Hierarchical clustering based on the distance between cells identified five astrocyte subclusters (AST1–5) with unique molecular and functional characteristics. During microglia subclustering, we chose 0.3 as the clustering resolution and applied FindAllMarkers() to identify the differential genes among cell subclusters, which combined with their potential biological functions, and finally achieved five different microglia subclusters (Micro1–5). Incidentally, oligodendrocyte lineage cells were further classified into five cellular subpopulations of different transcriptional states including oligodendrocyte progenitor OPCs, mature oligodendrocyte mOli, immature oligodendrocyte imOli, and two oligodendrocyte subclusters, Oli1 and Oli2, for subsequent analyses, utilizing the Seurat dimensionality-decreasing clustering procedure in conjunction with the classical cellular state marker genes.

### 4.4. Comparing Transcriptome Similarities Across Regional Cell Types and Subpopulations

To compare the similarity of cell-type transcriptomes in the temporal cortex and hippocampus, we utilized the spearman correlation of cell-type-specific expressed gene expression to assess cellular status in both regions. The FindAllMarkers() function was initially utilized to calculate the highly variable genes among cell types and neuronal subtypes in the temporal lobe cortex and hippocampus, respectively, and to extract the set of genes characterizing the cell types or subpopulations that are common to the two regions, which reduces the noisy interference of non-marker genes and improves the specificity. Next, the correlation between the expression profiles of subsets of corresponding cell type features in the two brain regions was assessed. High correlations represent transcriptomic similarities across corresponding cell types or subtypes in the temporal cortex and hippocampus, revealing consistency in cellular states. Cross-region comparisons (cortex vs. hippocampus) were performed exclusively using correlation analysis of cell-type-specific marker gene expression profiles, rather than co-clustering, thereby minimizing technical batch confounding.

### 4.5. Hotspot Analysis of Gene Coexpression

Hotspot is an advanced tool designed for the identification of highly correlated gene modules within single-cell datasets [17]. By leveraging the power of local autocorrelation, Hotspot precisely computes gene modules by isolating informative genes that exhibit significant spatial clustering. This tool evaluates pairwise correlations among genes, subsequently organizing the results into a gene-gene affinity matrix. Epilepsy-associated functions (EAF) categories were derived from Gene Ontology terms enriched in Hotspot co-expression modules. The 8 categories (cell adhesion, cell junction organization, development, differentiation, metabolic process, signaling, system process, transport) were curated by manually grouping significantly enriched GO terms (adjusted *p* < 0.05) based on semantic similarity.

### 4.6. Score According to Neuronal Activation and Functional Gene Sets

To quantify neuronal activation and assess epilepsy-related functional modules, we employed the AddModuleScore() method. This approach computes module scores that gauge the expression levels of specific gene sets pertinent to neuronal activity and epilepsy [19]. The neuronal activation marker gene set was meticulously curated from literature, ensuring it encompassed genes intricately involved in signaling pathways indicative of neuronal excitation. Concurrently, the functional gene set was systematically derived from the comprehensive compilation of genes associated with the corresponding GO term.

### 4.7. Reconstruction Pseudo-Time Trajectory by Monocle3

To discover the developmental transition of neuronal cell subtypes in the epileptic temporal cortex and hippocampus, we constructed pseudotemporal trajectories with the R package monocle3 (v1.4.26) [58]. Firstly, the docking of Seurat objects with monocle objects was implemented, the UMAP built-in fast stochastic gradient descent method was used to reduce the dimension, and the cells were visualized with each parameter defined by Seurat. Subsequently, cells were sorted according to their progression through the developmental program, and we chose to programmatically specify the root of the trajectory, with the excitatory neuron L6 subtype in the temporal lobe cortex as the initiating point, the CA3_Ex subtype, which is highly correlated with the L6 subtype, as the origin in the hippocampus, and the inhibitory neuron trajectory starting point for the cells in the In_SST and PVALB subtypes, respectively, for constructing the developmental trajectory, separately. Finally, genes that varied on the trajectory were identified according to the difference analysis method of graph autocorrelation analysis (graph_test). In the temporal cortex dataset, we sorted the genes according to their effect sizes on the trajectories and extracted the genes with q_value < 0.05 and morans_I > 0.5 as trajectory-associated genes, which were visualized with heatmaps. In the hippocampus dataset, we invoked find_gene_modules() to group the genes that changed on the trajectory into modules, and the heatmap shows the aggregated expression of all genes in each module for all subpopulations, we extracted the genes in the 3 modules with the highest expression in the cellular subpopulations respectively, which were considered to be trajectory-associated genes.

Sensitivity analysis of pseudotime root selection was performed by manually specifying alternative root cell types (e.g., L4_L5_6_RORB, L2_3_CUX2 for cortex; CA1_Ex, DG_Ex1 for hippocampus) using the same order_cells function. Pearson correlations between pseudotime and gene expression were computed using the ggpubr::stat_cor function.

### 4.8. Construction of Cross-Regional Cell Type-Specific Transcriptional Regulatory Networks

We employed the pySCENIC pipeline [59] to reconstruct cell type-specific transcriptional regulatory networks in the temporal lobe cortex and hippocampus based on co-expression and motif analysis, separately. Initially, GRNBoost was applied to infer transcription factors and candidate target gene modules based on co-expression; next, RcisTatget was carried out for TF-motif enrichment to identify regulators; in the end, AUCelll was computed to calculate the activity of regulators (regulons). We operated the calcRSS() function to extract the top five cell type-specific regulators and downloaded from published studies [60,61] and the AnimalTFDB v4.0 database (https://guolab.wchscu.cn/AnimalTFDB4/#/, accessed on May 2024) the list of known transcription factors that have been experimentally confirmed human transcription factors, with which we screened our inferred transcription factors to ensure the reliability of our inferred transcriptional regulatory network. We filtered the target genes of the regulators and functionally enriched the target genes (*p* value < 0.01) to exclude the genes not involved in the biological process, from which we obtained reliable regulators to construct a transregional cell-type specific transcriptional regulatory network. The network graph was visualised using Cytoscape (V. 3.10.4).

### 4.9. Downloading and Processing of Bulk RNA Data

Bulk RNA-seq datasets analyzed in this study, including GSE256068 (79 epilepsy patients versus 17 controls), GSE139914 (8 epilepsy versus 39 controls), and GSE140393 (12 epilepsy versus 9 controls), were sourced from the Gene Expression Omnibus (GEO) database. During data annotation, gene expression levels were assigned by averaging probe-level signals when multiple probes corresponded to identical gene symbols.

### 4.10. Differential Expression Analysis

Differential expression analysis of the epilepsy RNA-seq dataset GSE256068 (epilepsy versus controls) was conducted using the limma package [62], implementing empirical Bayes moderation of standard errors. Significantly differentially expressed genes (DEGs) were defined by thresholds of |log_2_(fold change)| > 1 and adjusted *p*-value (adj.P.Val) < 0.05.

### 4.11. Identification of Hub Genes and Development of a Diagnostic Signature for Epileptic Neuronal Activation

Hub genes were identified based on differentially expressed genes from the GSE256068 dataset and target genes from neuron activation-related transcriptional modules. Hub genes were prioritized through integrated feature selection using least absolute shrinkage and selection operator (LASSO) regression and recursive feature elimination (RFE) coupled with random forest. LASSO analysis, implemented via the glmnet (v4.1-9) [63] R package with 10-fold cross-validation, selected the optimal penalty parameter (λ) by minimizing binomial deviance. Concurrently, RFE analysis (caret package [64]) employed random forest-based gene ranking with sequential evaluation of feature subsets (1–10, 15, 20 genes) under 10-fold cross-validation, maximizing classification accuracy. The intersection of LASSO and RFE outputs identified consensus hub genes. A logistic regression diagnostic model was subsequently constructed using these hub genes. Model performance was quantified via receiver operating characteristic (ROC) analysis using the qROC package, with AUC values computed for both the integrated model and individual hub genes. External validation was performed on independent datasets (GSE139914, GSE140393) using identical analytical pipelines.

### 4.12. Enrichment Analyses

For the purpose of mapping Gene Ontology (GO) biological process descriptions to each major cell type, identifying genetically relevant functions in neuronal cell developmental trajectory trajectories, and characterizing the biological functions of neuroglia that play a predominant role in a hippocampal organization, we applied the enrichGO() in the R package clusterProfiler (version 4.4.4) for gene set enrichment analysis, the threshold was set according to the default parameters, the *p*-value was adjusted using FDR, and enrichment was considered to exist at *p*-value < 0.05. GO terms were ranked by *p*-value and the top 5 identified GO terms shared between cell types were selected and visualized using the R library BPG [65] (v7.0.5). Additionally, the R package ComplexUpset was used to visualize the unique and shared biological functional entries that the three major classes of glial cells have at the anterior and posterior ends of the hippocampus.

### 4.13. Inference of Regulatory Relationships Among Genes in Enriched Pathways

The R package aPEAR exploits the similarities between sets of pathway genes to aggregate pathways into modules, each highlighting the most prominent biological functional terms, which we utilize to simplify the important functions involved in the developmental trajectories of neurons in the hippocampus. To gain insight into important molecular functions in developmental trajectories in inhibitory neurons in the temporal lobe cortex, we also utilized the R package CBNplot [66] to infer gene-gene interactions as well as upstream and downstream regulatory relationships based on the gene’s expression level across cells. Moreover, the R package ggsankey was used to clearly demonstrate the relationships between key biological functions and the inclusion of genes enriched to flow among them.

### 4.14. Statistics

All graphical constructions in this study were performed using R (v4.2.2) software. The Wilcoxon rank-sum test was used for pairwise comparisons of gene expression levels (differential expression analysis). For cell-type proportion data, we used scCODA, as described in Section 4.2. The *p* < 0.05 was defined as statistically significant.

## Data Availability

The data that support the findings of this study are openly available in GEO at GSE160189 (https://www.ncbi.nlm.nih.gov/geo/query/acc.cgi?acc=GSE160189, accessed on 10 September 2023) and GitHub (https://github.com/khodosevichlab/Epilepsy19, accessed on 15 October 2023). Bulk RNA-seq datasets analyzed in this study, including GSE256068 (https://www.ncbi.nlm.nih.gov/geo/query/acc.cgi?acc=GSE256068, accessed on 15 August 2024), GSE139914 (https://www.ncbi.nlm.nih.gov/geo/query/acc.cgi?acc=GSE139914, accessed on 15 August 2024), and GSE140393 (https://www.ncbi.nlm.nih.gov/geo/query/acc.cgi?acc=GSE140393, accessed on 15 August 2024), were sourced from the Gene Expression Omnibus (GEO) database. All analyses were performed using R (v4.2.2) and Python (v3.9). The complete set of custom scripts used for data processing, analysis, and figure generation is available at https://github.com/Zhangyunpeng1987/Epilepsy (accessed on 6 May 2026). For detailed parameter settings, please refer to the annotated scripts and the README file.

## Supplementary Materials

The following supporting information can be downloaded at https://www.mdpi.com/article/10.3390/ijms27104466/s1.
