# Supplementary material for: Uncovering the Key Circuit FOSL2/FOS/EGR3/EGR1, Contributing to the Hyperexcitability of Excitatory Neurons in the Epileptic Temporal Cortex and Hippocampus
Source: Int J Mol Sci. 2026 May 16;27(10):4466. doi: 10.3390/ijms27104466 (PMC13206787; doi:10.3390/ijms27104466)
Supplement: Supplementary file 1 [file ijms-27-04466-s001.zip › Supplementary Figures.pdf]

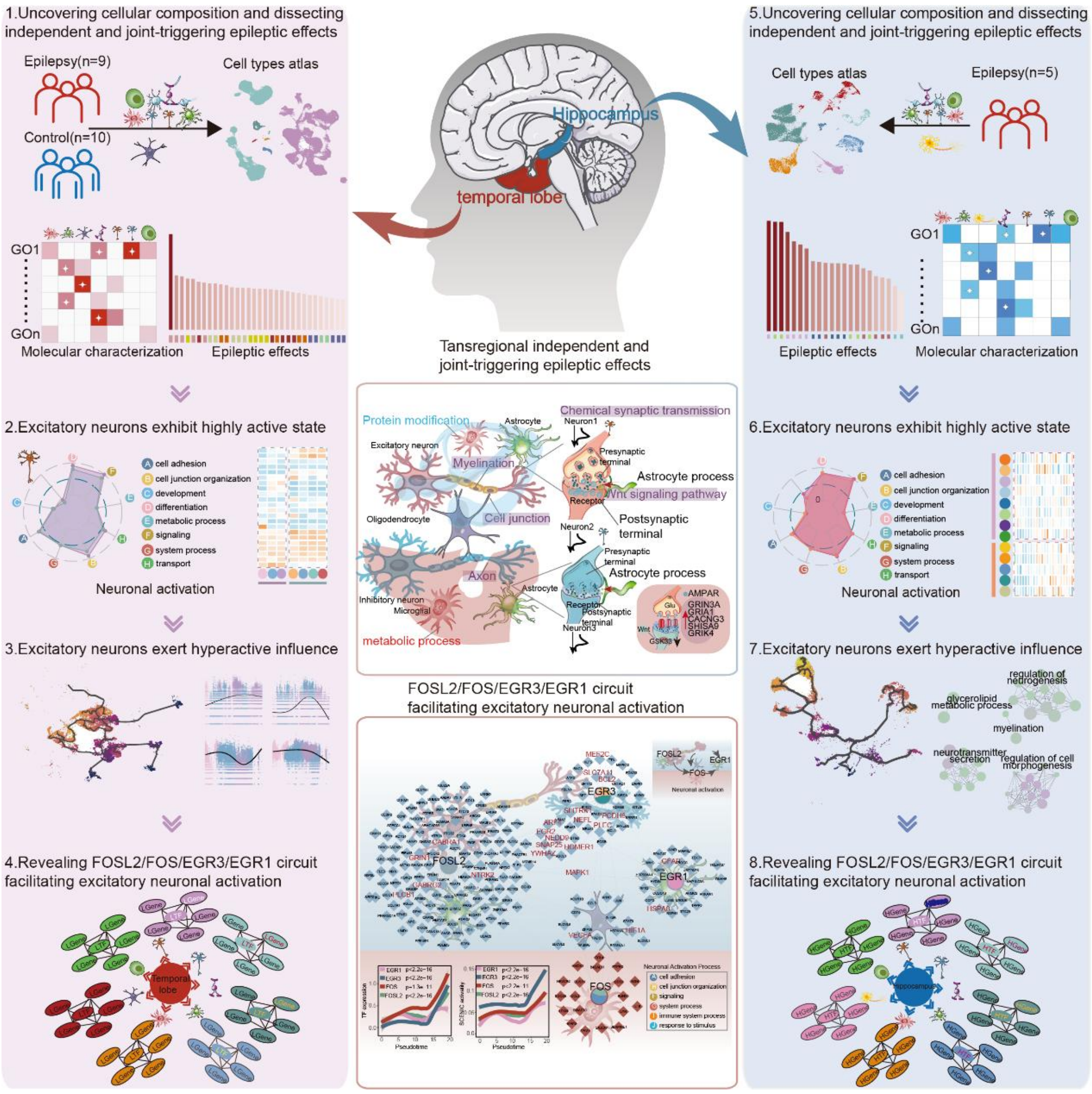

Figure S1. Analytical workflow diagram

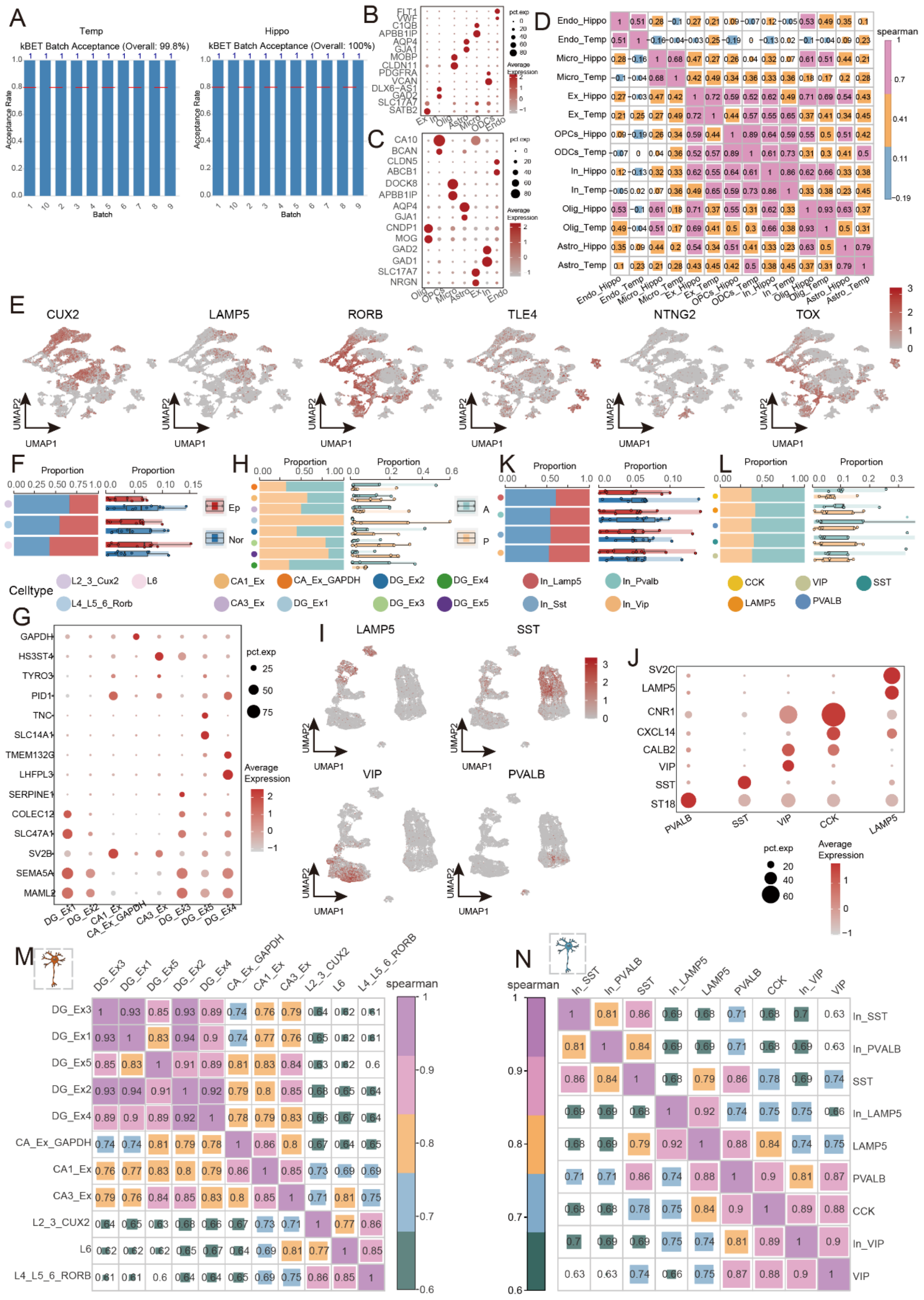

Figure S2. Cellular diversity and heterogeneity in the temporal lobe and hippocampus of the human epilepsy brain. (A) kBET acceptance rates per batch for the temporal cortex (left) and hippocampus (right). (B, C) Expression of marker genes annotated for major cell types in the temporal lobe (B) and hippocampus (C). Ex, excitatory neurons;

In, inhibitory neurons; Astro, astrocytes; Micro, microglia; Olig, oligodendrocytes; ODCs, oligodendrocyte precursor cells; OPCs, oligodendrocyte progenitor cells; Endo, endothelial cells. (D) Spearman's correlation heatmap shows the transcriptomic correspondence relations of the major cell types identified in the temporal cortex and hippocampus. (E). Representative genes of excitatory neuron subtypes in the temporal lobe cortex. (F) Bar graph showing the proportion of excitatory neurons in the temporal cortex. (G) Representative genes of excitatory neuron subtypes in the hippocampus. (H) Bar graph showing the proportion of excitatory neurons in the hippocampus. (I, J). Representative genes of inhibitory neuron subtypes in the temporal lobe cortex(I) and hippocampus(J). (K, L) Bar graph showing the proportion of inhibitory neurons in the temporal cortex(K) and hippocampus(L). (M, N) Correlation heatmap demonstrates transcriptomic correspondence of excitatory(M) and inhibitory neuronal(N) subtypes across the temporal lobe cortex and hippocampus.

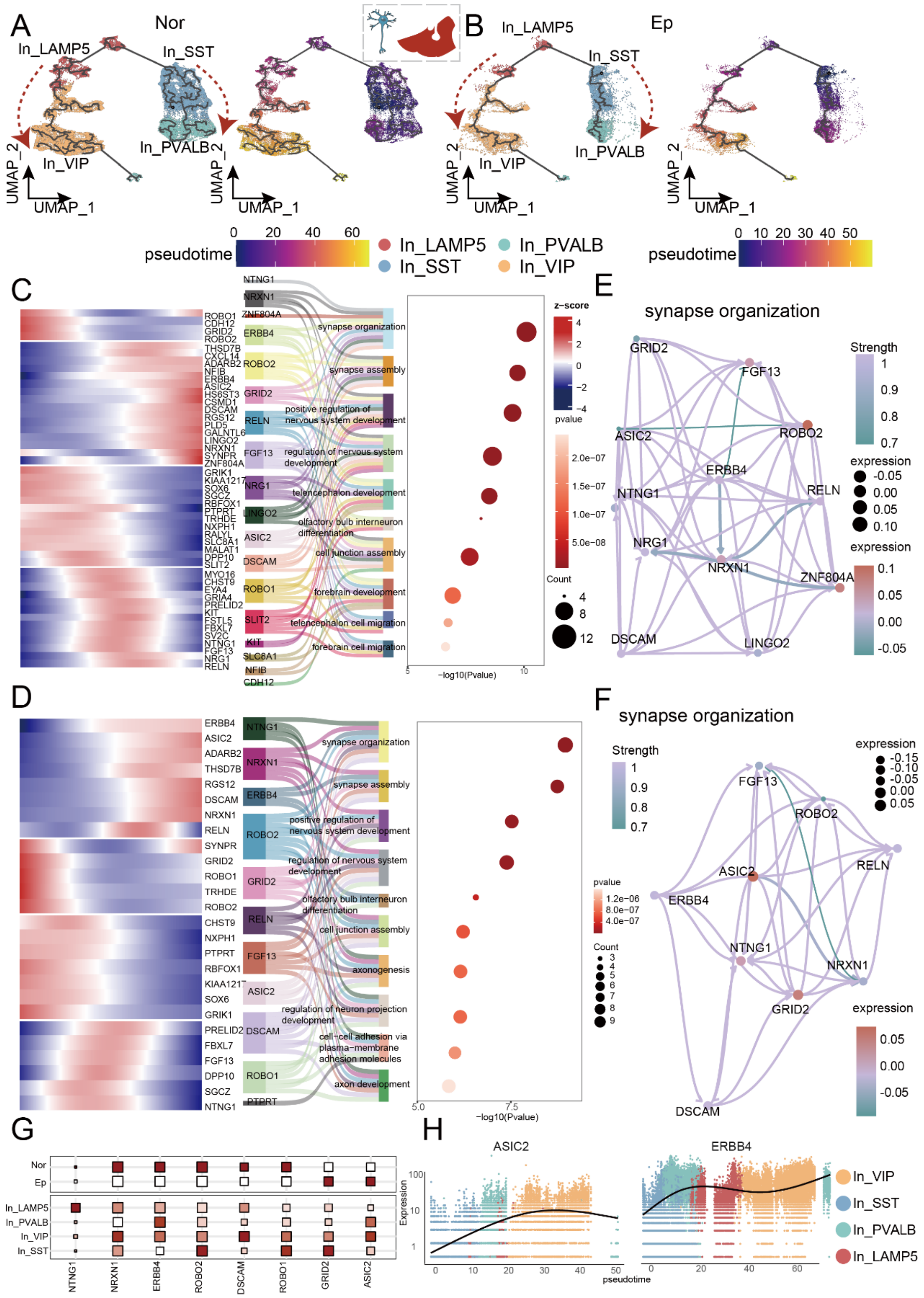

Figure S3. Dynamic shifts in temporal lobe inhibitory neuron subtypes in disease and normalcy. (A, B) pseudo-time developmental trajectories of inhibitory neurons in non-epileptic (A) and epileptic (B) temporal lobes. Cells are colored according to cell identity (left) and pseudotime (right), respectively. (C) Transcriptional transitions of

inhibitory neurons in the normal temporal lobe along pseudotime. Left: Heatmap illustrates gene expression associated with cell fate decisions. The rows (genes) of the heatmap are clustered and the columns (cells) are sorted over time. Different stages are labeled with various colors. Middle: The Sankey plot shows the biological processes in which the genes on the left, with different colors pointing to different genes. Right: The dot plot highlights the significance level of the biological processes. (D) Predicted regulatory relationships between internal genes involved in synaptic organization in normal temporal cortex. The shade of the line represents the strength of the interactions, with the redder color of the dot representing higher expression of the gene. (E) Transcriptome alterations during the development of epileptic temporal lobe inhibitory neurons. Left: Heatmap shows changes in the expression of genes essential for the developmental trajectory. Middle: Sankey diagram demonstrates biological processes of trajectory genes. Right: The dot plot displays the significance level of GO terms. (F) Predicted regulatory relationships between internal genes involved in synaptic organization in the epileptic temporal cortex. The shade of the line represents the strength of the interactions, with the redder color of the dot representing higher expression of the gene. (G) Dot plot illustrates the expression levels of inhibitory neuron cell fate important genes in various cell subtypes and distinct tissues. (H) Jitter plot showing the expression of genes activated in the developmental trajectory.

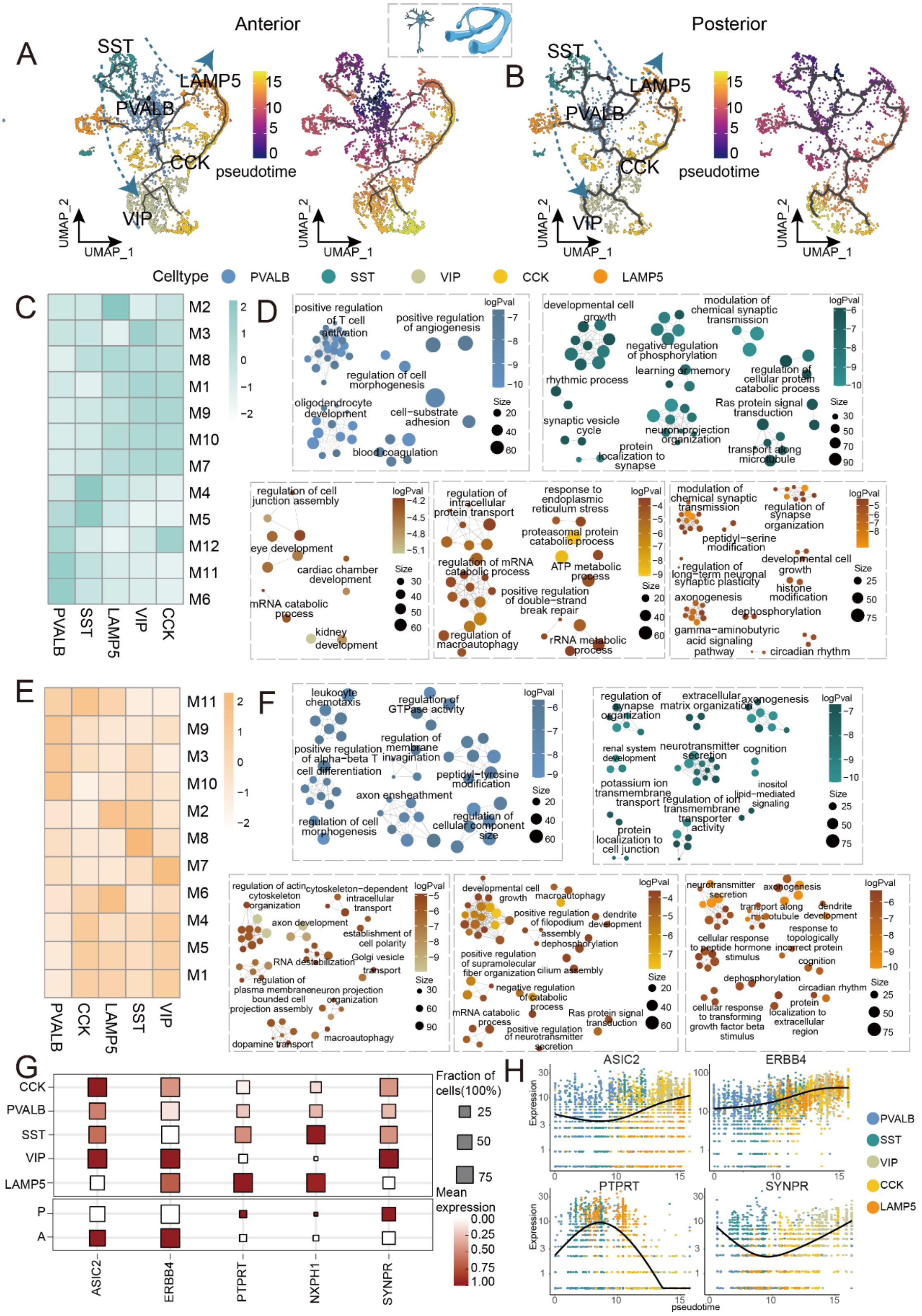

Figure S4. Dynamic differentiation trajectories of inhibitory neuron subtype diversity in hippocampus. (A, B) inhibitory neuron subtypes in anterior (A) and posterior (B) hippocampus pseudotime traces. Cells are colored according to cell identity (left) and pseudotime (right), separately. (C) Co-expression heatmap shows cell type-specific as

well as shared modules in the anterior hippocampus, where each module displays the aggregated expression values of genes changed along the developmental trajectory involved in each cell subtype. (D) Functional network diagram of the top 50 GO entries enriched for genes of the three modules with the highest expression in each anterior hippocampus cell subpopulation. A box qualifies a cell subtype and the functional color matches the cell type color. (E) Heatmap of coexpression for inhibitory neuron subtypes in the posterior hippocampus demonstrates trajectory-related specificity to cell type as well as shared modules. (F) Functional network map of trajectory-related genes for each inhibitory neuron subpopulation in the posterior hippocampus. (G) Dot plots display the expression of genes associated with functional enrichment and trajectory-related modules of inhibitory neuron cells in different cell subtypes and in different positions in the hippocampus. (H) Expression dynamics of genes activated in developmental trajectories along pseudo-time trajectories.

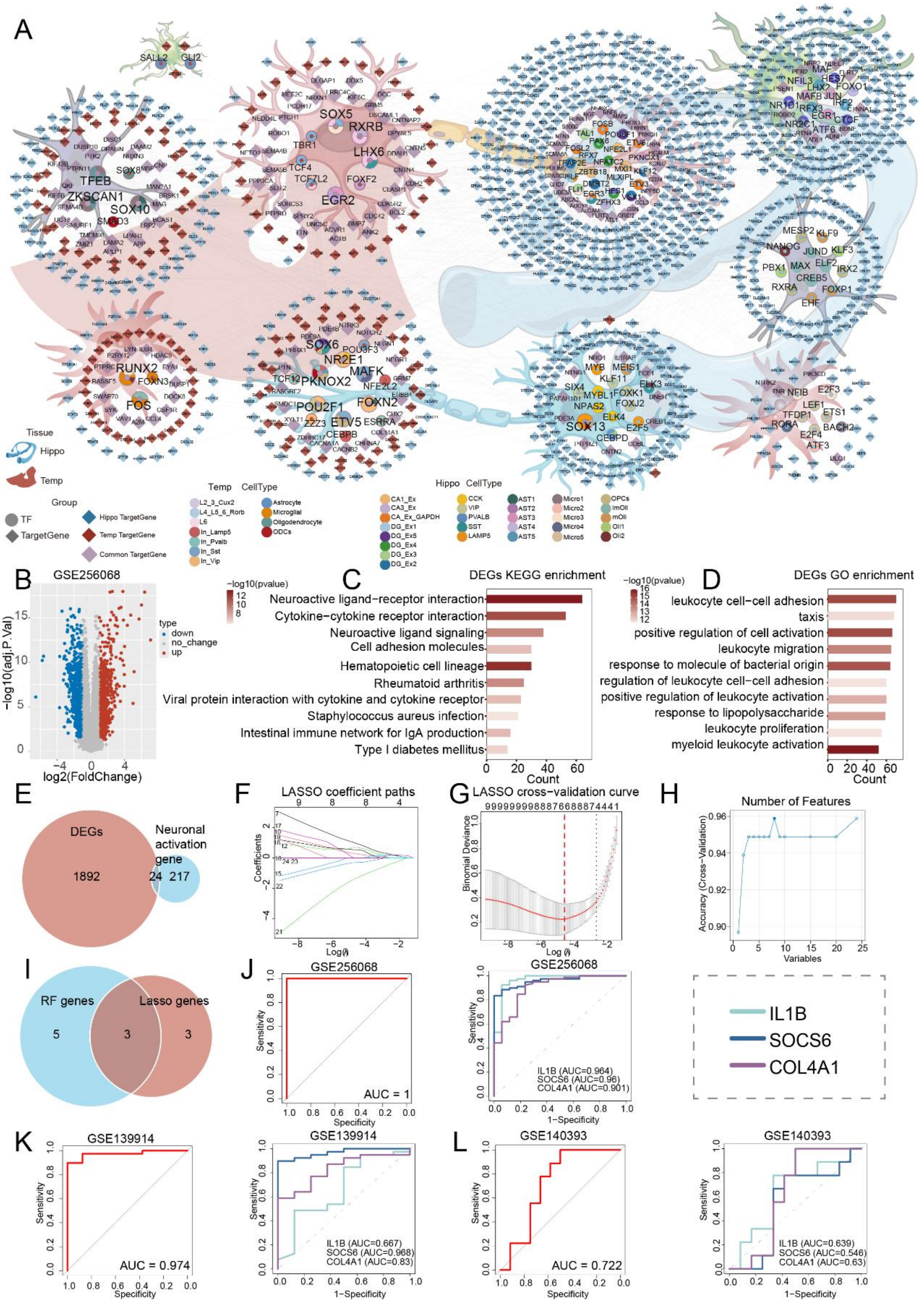

Figure S5. Transcriptional networks and diagnostic signature development. (A) Illustrated are cell type-specific transcriptional regulatory networks in the epileptic temporal cortex (left) and hippocampus (right). Circles represent transcription factors (TFs), with inner pie chart colors denoting cell subtypes. Diamonds mark target genes

(TargetGene) bound by TFs. Purple diamonds denote target genes present in both regions, red for the temporal lobe, and blue for the hippocampus. The shaded areas below depict distinct tissues: blue for the hippocampus, red for the temporal cortex, with various shapes denoting different cell types. Abbreviations: TF, Transcription factor; Temp, temporal cortex; Hippo, hippocampus. (B) Volcano plot of differentially expressed genes (DEGs) in epilepsy versus controls (GSE256068). Red/blue dots denote up/downregulated genes ($|\log_2 FC| > 1$, adj.P.Val < 0.05). (C-D) Functional enrichment of DEGs in GO biological processes (C) and KEGG pathways (D). (E) Intersection analysis identifying 24 genes overlapping between DEGs and targets of the neuronal activation transcriptional circuit (FOSL2/FOS/EGR3/EGR1). (F) LASSO coefficient trajectories. (G) Cross-validated error minimization for optimal λ selection. (H) Recursive feature elimination (RFE) accuracy versus gene subset size. (I) Venn diagram showing three consensus hub genes (IL1B/SOCS6/COL4A1) from LASSO-RFE integration. (J-L) ROC curves for the diagnostic model and individual hub genes in training (J: GSE256068) and validation cohorts (K: GSE139914; L: GSE140393).

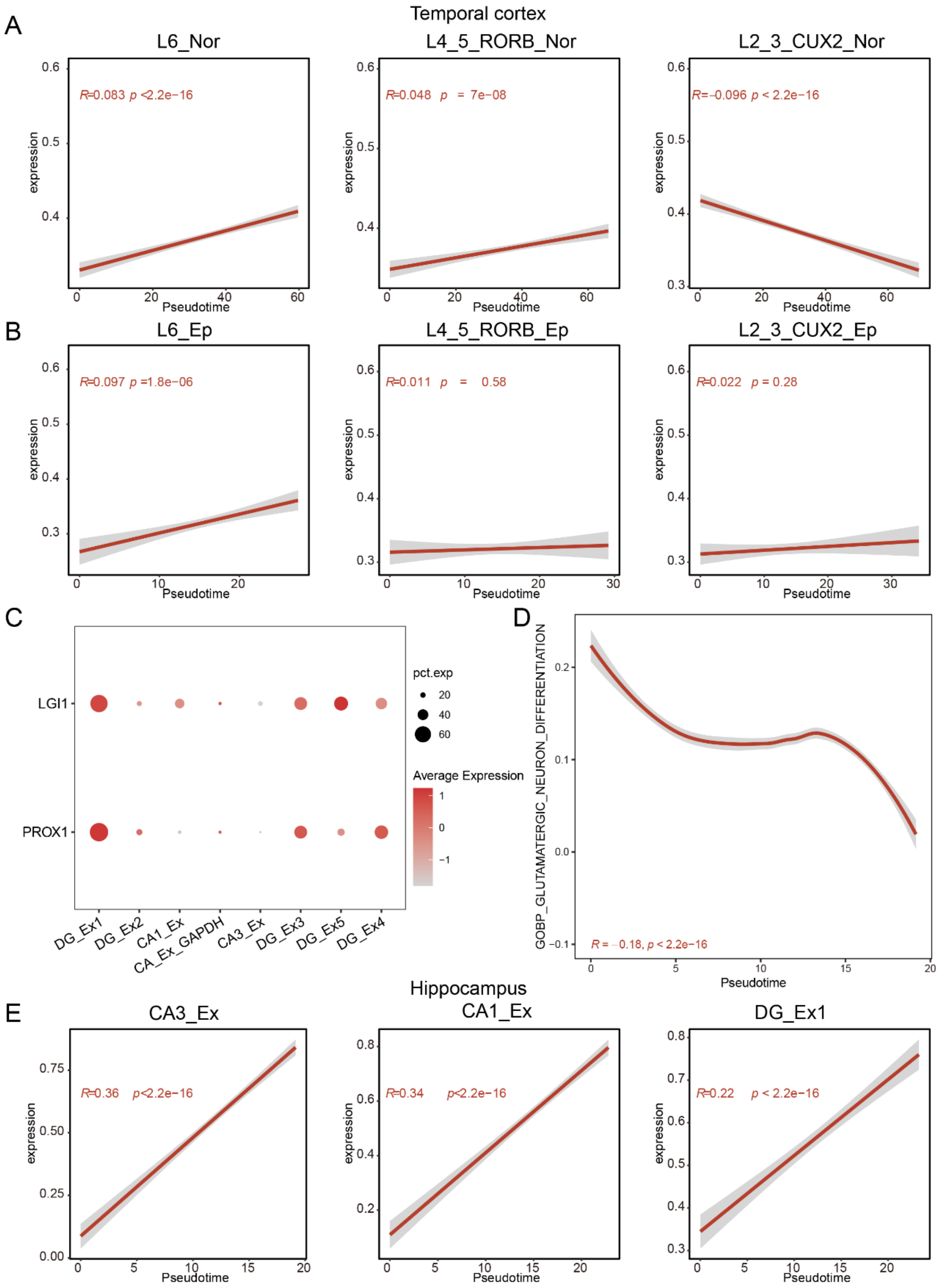

Figure S6. Sensitivity analysis of pseudotime root choice. (A) Temporal cortex (non-epileptic): average expression of FOSL2/FOS/EGR3/EGR1 versus pseudotime under three root assignments (L6, L4_L5_6_RORB, L2_3_CUX2). (B) Temporal cortex (epileptic): same as (A). (C) Dot plot of PROX1 and LGI1 expression across hippocampal excitatory subtypes, illustrating that these maturation markers are lowest

in CA3_Ex and highest in DG_Ex1. (D) Module score of GOBP_GLUTAMATERGIC_NEURON_DIFFERENTIATION along pseudotime (root = CA3_Ex), showing a significant negative correlation, indicating decreasing differentiation potential with pseudotime. (E) Hippocampus: average FOSL2/FOS/EGR3/EGR1 expression versus pseudotime with roots set to CA3_Ex, CA1_Ex, or DG_Ex1.
